# Emerging Roles of 1D Vertical Nanostructures in Orchestrating Immune Cell Functions

**DOI:** 10.1002/adma.202001668

**Published:** 2020-08-26

**Authors:** Yaping Chen, Ji Wang, Xiangling Li, Ning Hu, Nicolas H. Voelcker, Xi Xie, Roey Elnathan

**Affiliations:** ^1^ Monash Institute of Pharmaceutical Sciences Monash University 381 Royal Parade Parkville VIC 3052 Australia; ^2^ Melbourne Centre for Nanofabrication Victorian Node of the Australian National Fabrication Facility 151 Wellington Road Clayton 3168 Australia; ^3^ The First Affiliated Hospital of Sun Yat‐sen University Sun Yat‐sen University Guangzhou 510006 China; ^4^ State Key Laboratory of Optoelectronic Materials and Technologies School of Electronics and Information Technology Sun Yat‐sen University Guangzhou 510006 China; ^5^ Department of Materials Science and Engineering Monash University 22 Alliance Lane Clayton VIC 3168 Australia; ^6^ Commonwealth Scientific and Industrial Research Organisation (CSIRO) Clayton VIC 3168 Australia; ^7^ INM‐Leibniz Institute for New Materials Campus D2 2 Saarbrücken 66123 Germany

**Keywords:** 1D vertical nanostructures, immune cells, immunomodulation, intracellular delivery, nano–bio interface

## Abstract

Engineered nano–bio cellular interfaces driven by 1D vertical nanostructures (1D‐VNS) are set to prompt radical progress in modulating cellular processes at the nanoscale. Here, tuneable cell–VNS interfacial interactions are probed and assessed, highlighting the use of 1D‐VNS in immunomodulation, and intracellular delivery into immune cells—both crucial in fundamental and translational biomedical research. With programmable topography and adaptable surface functionalization, 1D‐VNS provide unique biophysical and biochemical cues to orchestrate innate and adaptive immunity, both ex vivo and in vivo. The intimate nanoscale cell–VNS interface leads to membrane penetration and cellular deformation, facilitating efficient intracellular delivery of diverse bioactive cargoes into hard‐to‐transfect immune cells. The unsettled interfacial mechanisms reported to be involved in VNS‐mediated intracellular delivery are discussed. By identifying up‐to‐date progress and fundamental challenges of current 1D‐VNS technology in immune‐cell manipulation, it is hoped that this report gives timely insights for further advances in developing 1D‐VNS as a safe, universal, and highly scalable platform for cell engineering and enrichment in advanced cancer immunotherapy such as chimeric antigen receptor‐T therapy.

## Graphical abstract

1D vertical nanostructures (1D‐VNS) are used in immunomodulation and intracellular delivery into immune cells. 1D‐VNS produce biophysical and biochemical signals to modulate cellular deformation, membrane tension, and immune activation cascades; they facilitate efficient intracellular delivery of diverse therapeutic cargoes into immune cells.

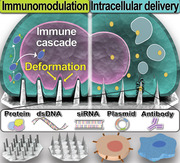

## 1 Introduction

Advances in bionanotechnology have fostered significant improvements in biomedical engineering and preclinical studies.^[^ ^1^ ^]^ Impressive progress in the nascent field of programmable nano–bio interfaces—especially in the design of nanotopographies optimized to facilitate biomolecular delivery, genome editing, and cellular modulation—have enormous potential to build capacity over a range of collaborating disciplines associated with biomedical research.

**Figure 1:**
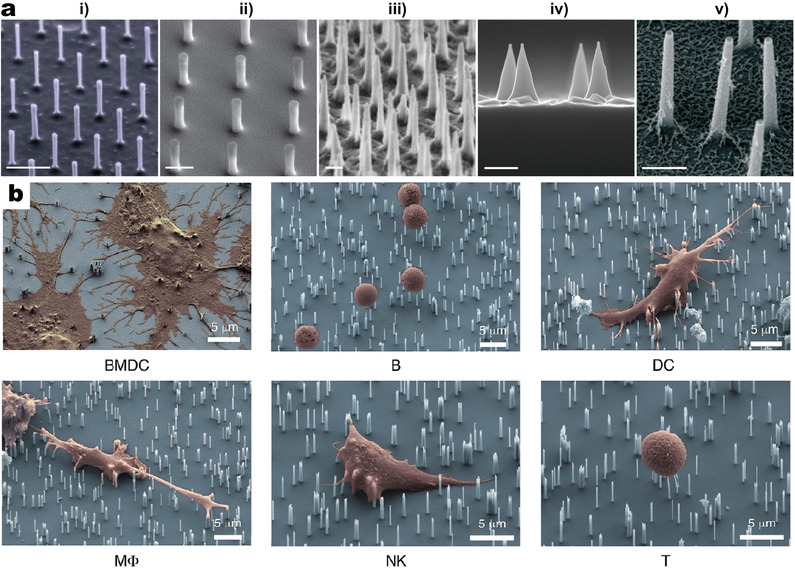
Emerging application of 1D‐VNS platforms in immunological studies. a) SEM images of five types of 1D‐VNS, including nanowires (i), nanopillars (ii), nanoneedles (iii), nanocones (iv), and nanostraws (v). Scale bars: 1 µm (i–iii) and 500 nm (iv,v). i) Reproduced with permission.^[^ ^6^ ^]^ Copyright 2010, The Authors, published by National Academy of Sciences; ii) Reproduced with permission.^[^ ^7^ ^]^ Copyright 2015, Springer Nature; iii) Reproduced with permission.^[^ ^30^ ^]^ Copyright 2015, ACS; iv) Reproduced with permission.^[^ ^31^ ^]^ Copyright 2018, Elsevier; v) Reproduced with permission.^[^ ^32^ ^]^ Copyright 2016, ACS. b) SEM images of primary mouse bone‐marrow dendritic cells (BMDCs), B cells, dendritic cells (DCs), macrophages (MΦs), natural killer (NK) cells, and T cells (false colored orange) on top of SiNWs (false colored blue) after 24 h culture. Reproduced with permission.^[^ ^28^ ^]^ Copyright 2014, ACS.

High‐aspect‐ratio nanostructures are now providing major advantages in precise manipulation of increasingly complex cellular processes, assisting the translation into clinical applications such as tissue engineering, regenerative medicine, drug delivery, biosensing, and cancer immunotherapies.^[^ ^2^, ^3^, ^4^, ^5^ ^]^ In particular, 1D vertical nanostructures (1D‐VNS)—nanowires, nanoneedles, nanopillars, nanotubes, nanosyringes, nanostraws, nanocones, and nanospikes (**Figure** **1**a)—have helped tackle various biological problems such as intracellular recording and genetic interrogation.^[^ ^6^, ^7^, ^8^, ^9^, ^10^, ^11^, ^12^, ^13^, ^14^, ^15^, ^16^ ^]^ Unlike other high‐aspect‐ratio nanostructures, such as freestanding carbon nanotubes, 1D‐VNS can be rationally designed and synthesized, via top‐down and/or bottom‐up approaches, with defined key parameters—including topological geometry (pitch, diameter, length), chemical composition, doping, and electronic properties.^[^ ^17^, ^18^, ^19^, ^20^, ^21^ ^]^ So by well‐controlled programming coupled with selective surface functionality, 1D‐VNS can provide unprecedented spatial and mechanical resolution to enable direct, sensitive, and rapid analysis of biological species and manipulation of cellular activities. For instance, silicon nanowires (SiNWs) with nanoscale diameter (1–100 nm) and high aspect ratio (<10^3^) enable direct access into intracellular compartments of living cells, which can greatly facilitate the study of complex regulatory and signaling pathways at cellular and subcellular levels.^[^ ^22^ ^]^ A wide variety of mammalian cells (adherent and nonadherent, cell lines and primary cells) have been investigated to probe biosystems using 1D‐VNS platforms. Significant work has recently been done on immune cells—the central players in disease fighting that hold great clinical potential.

Early attempts to modulate nanomaterial‐immune system interactions by tuning the topographical characteristics and/or surface chemical properties of nanomaterials focused on macrophages, an innate immune cell type involved in nonspecific host defense against pathogen infections.^[^ ^23^, ^24^, ^25^ ^]^ Research interest has since widened to embrace different cell types from the vast immunity family, covering both innate immune cells such as monocyte, dendritic cell (DC), nature killer (NK), neutrophil, and adaptive immune cells such as T and B lymphocytes (Figure 1b).^[^ ^26^, ^27^, ^28^, ^29^ ^]^

The major advantage of 1D‐VNS materials arises from their nanoscale dimensions: enhanced interaction with immune cells, which allows bidirectional information flow to be regulated by biophysical and biochemical signals traveling both into and out of immune cells. VNS‐induced membrane deformation and rearrangement of local cellular components, such as actin networks and membrane‐associated proteins, may lead to a cascade of immune responses. VNS‐displaying platforms, including planar substrates^[^ ^9^, ^23^, ^24^, ^33^ ^]^ and 3D microparticles,^[^ ^34^ ^]^ have demonstrated the ability to modulate in vitro and in vivo immune responses. Provided with localized proengraftment cues, nanopatterned bulk metallic glasses triggered the release of inflammatory factors;^[^ ^9^ ^]^ micro‐ and nanopatterned topographical cues modulated macrophage cell shape and phenotype;^[^ ^24^ ^]^ PDMS nanopillars amplified cytotoxic CD8^+^ T lymphocyte (CTL) response by stimulating the formation of actin‐rich protrusions;^[^ ^33^ ^]^ and nanospike‐decorated TiO_2_ microparticles activated DCs by upregulating expression of costimulatory molecules like CD40.^[^ ^34^ ^]^

Recent success in VNS‐mediated intracellular delivery suggests that 1D‐VNS represent a promising platform for cell‐based immunotherapy by introducing exogenous genetic and therapeutic materials into immune cell candidates.^[^ ^6^, ^27^, ^28^, ^35^ ^]^ VNS allows rapid and direct intracellular access while inducing minimal cell cytotoxicity; a wide range of biomolecules, including DNAs, RNAs, proteins, quantum dots (QDs), and impermeable drugs, have been successfully delivered into live cells.^[^ ^6^ ^]^ In particular, SiNW arrays demonstrated high efficiency (>90%) in transfecting exogenous genetic materials into primary immune cells—which are notoriously hard to transfect—while maintaining high viability and immunological competence;^[^ ^27^, ^28^, ^36^ ^]^ siRNAs were transfected via SiNWs into primary B cells and CD4^+^ T cells to knock down predicted target genes, facilitating discovery of essential signaling pathways and genetic regulatory circuits for immune cell activation and differentiation;^[^ ^27^, ^28^ ^]^ SiNWs were used to administer a small‐molecule inhibitor of the enzyme Polo‐like kinase (Plk) into bone‐marrow derived dendritic cells (BMDCs), confirming the essential role of Plk2 and Plk4 in regulating antiviral gene expression in these cell;^[^ ^37^ ^]^ by using vertical carbon nanosyringe arrays (CNSAs) under applied centrifugal g‐force, pEGFP plasmids were transfected into primary lymphocytes with significantly higher efficiency than conventional Lipofectamine 2000‐mediated method.^[^ ^38^ ^]^

Despite the emerging use of 1D‐VNS technology in immunology, a deeper understanding of VNS–immune cell interplay is urgently needed, especially on how direct physical entanglement with VNS and indirect mechanotransduction alter immune cell status, and how VNS interfacing mechanisms could be the basis for immune cell engineering. Such understanding will help to develop strategies and solutions to overcome side effects in the use of 1D‐VNS devices, which still represent an important challenge in biomedical research. The progress report is a snapshot of this very active and dynamic area. We summarize the most recent studies on the performance of 1D‐VNS in orchestrating immune cell functions, behaviors, and fate conversions through experimental and theoretical studies. Specifically, two major aspects are examined: i) the effects of biophysical/biochemical cues from 1D‐VNS on triggering immune responses and modulating functions of different immune cell types; and ii) the mechanisms and efficacy of VNS‐mediated intracellular delivery for immune cells. By identifying fundamental challenges and exploring opportunities for further interdisciplinary research into VNS‐mediated immune‐cell manipulation and interrogation, we expect the development and use of 1D‐VNS to be instrumental in meeting biomedical goals.

## 2 Application of Nanotechnology in Immunomodulation

### 2.1 Immune System Network

**Figure 2:**
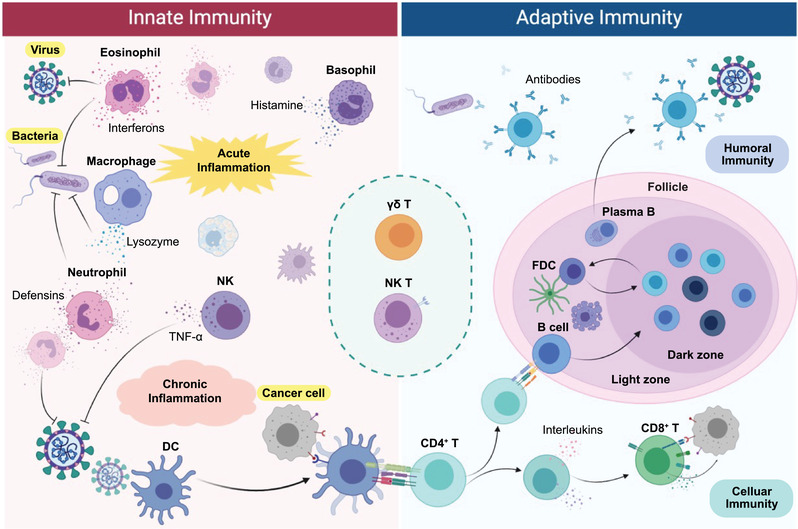
Schematic of the complex network of host immune system, comprising innate and adaptive immunity, to recognize and target non‐self‐pathogens, including virus, bacteria, and cancer cell (highlighted in yellow).

The immune system has evolved to elicit nonspecialized innate and highly specialized adaptive immune responses (**Figure** **2**) to distinguish self from nonself (e.g., viruses, bacteria, and cancer cells), eliminating nonself subjects from the body. Numerous types of cells are involved in the generation of effective immune responses against antigens.^[^ ^39^ ^]^ A good analogy to the molecular mechanisms of immune responses is that of an orchestral performance. Each musician has specialized skills and reads from a different set of music; but everyone is coordinated by the conductor. The processes that generate and regulate the host immunity are comparable: different arms of the immune system are coordinated by genes that encode for proteins with specific functions.

#### 2.1.1 Innate Immunity

Innate immune cells form the front line to defend nonspecific pathogenic threats that bypass natural physical and chemical barriers of the body. Neutrophils, monocytes, macrophages, basophils, eosinophils, DCs, and NK cells are key components of the innate system. They eliminate invading pathogens and cancer cells by various mechanisms, including inflammation, reactive oxygen species release, complement activation, receptor‐mediated killing, phagocytosis, and autophagy.^[^ ^40^ ^]^ Inflammatory mediators—such as histamine, defensins, lysozyme, chemokines, and cytokines like interferon (IFN) and tumor necrosis factor (TNF)‐α—are often secreted during acute inflammation to clear the initial cause of cell injury and tissue damage; classic symptoms accompanying inflammation include heat, pain, redness, swelling, and loss of function. But persistent acute inflammation may lead to a progressive shift in the local microenvironment, where destruction and healing of the tissue occur simultaneously; this results in chronic inflammation, which is associated with diseases such as asthma, atherosclerosis, and osteoarthritis.^[^ ^41^ ^]^

#### 2.1.2 Adaptive Immunity

When an innate immune response is insufficient to eradicate an infection, the adaptive immune system is informed by antigen presenting cells (APCs) such as DCs. Once mobilized, they recruit an army of immune cells—mainly T and B lymphoctyes—specifically designed to attack that antigen. CD4^+^ helper T cells are pivotal in adaptive immunity, since they are required for almost all adaptive immune responses, comprising cellular and humoral immunity.^[^ ^42^, ^43^ ^]^ In cell‐mediated immunity, CD4^+^ T cells release a spectrum of cytokines to activate cytotoxic CD8^+^ T (CTL) cells, promoting their differentiation and maturation to recognize, bind, and lyse infected target cells.^[^ ^44^, ^45^, ^46^ ^]^ Humoral immunity is mediated by antibodies generated by B cells. With the assistance from CD4^+^ T cells, B cells undergo clonal expansion, class switching, and somatic hypermutation to differentiate into plasma B cells that can produce high‐affinity antibodies against a specific antigen; these antibodies bind to antigens, neutralizing them, or causing lysis or phagocytosis.^[^ ^43^, ^47^ ^]^ Importantly, adaptive immunity forms a “memory”—sometimes life‐long—that makes it more efficient to evoke future responses against the same antigen.

#### 2.1.3 Immunomodulation in Therapeutic Interventions

Immunomodulation is the core of multiple therapeutic interventions, including but not limited to immunotherapies for tumors, infectious diseases, autoimmune diseases, and graft‐versus‐host diseases. Given that most applications proposed by 1D‐VNS studies were on cancer immunotherapy, the progress report is mainly focusing on this area.

Modulation of the immune system to enhance anticancer response holds great promise in the prevention and treatment of the reoccurrence and metastasis of cancer. Numerous modalities have been involved in immunotherapeutic translation, including cancer vaccines, oncolytic viruses, and administration of such immunomodulators as cytokines,^[^ ^48^ ^]^ monoclonal antibodies,^[^ ^49^ ^]^ and checkpoint inhibitors,^[^ ^50^ ^]^ which either costimulate cells or block the so‐called immune checkpoint pathways.^[^ ^51^ ^]^ Immunotherapeutic regimes that use specific monoclonal antibodies to block cytotoxic T lymphocyte‐associated protein 4 (CTLA‐4) and programmed cell death protein 1 (PD1) have shown exciting results for melanoma treatment.^[^ ^52^ ^]^ Cell‐based immunotherapy, such as adoptive cell transfer (ACT) of ex vivo activated T cells, is ideal for activating immune response, by generating effector T cells to recognize and kill tumor cells. In particular, chimeric antigen receptor (CAR)‐T cell therapy is one of the most promising approaches in treating hematological malignancies, such as relapsed or refractory B cell acute lymphoblastic leukemia and diffuse large B cell lymphoma.^[^ ^53^, ^54^ ^]^

Despite significant progress, clinical and commercial translation of laboratory‐based immunotherapy technologies is still stymied by unpredictable toxicity and immunogenicity, limited efficiency in treating large solid tumors, costly and lengthy procedure, insufficient in vitro expansion for scaled‐up production, and off‐target side effects from systemic dosing.^[^ ^55^, ^56^ ^]^

### 2.2 Effects of Biophysical Cues Induced by 1D‐VNS on Immune Cells

Due to the staggeringly complicated immune regulatory network, precise cues are needed to induce and fine‐tune the response of a specific cell type. Much work has studied how immune cells react to biochemical cues such as bacterial lipopolysaccharide (LPS), CpG DNA, viral RNA, and Toll‐like receptor (TLR) agonists.^[^ ^57^, ^58^, ^59^ ^]^ But a smaller body of work has examined the role of biomechanical and biophysical cues—such as envelope spike proteins on coronavirus, and pilus/fimbria on bacteria^[^ ^60^, ^61^, ^62^, ^63^ ^]^—in triggering host immunity. Indeed, biophysical cues have been increasingly recognized as critical regulators of cancer progression, detection biomarkers, and therapeutic targets. Cell‐based stiffness sensors can reveal how cells actually feel in their native environment and dynamically interrogate the mechanobiology of primary tumors and metastases. Existing immune cell therapies, such as CAR‐T cells, could be further engineered to exploit the biophysical cues of tumors to improve target specificity.

With programmed topological characteristics and surface functionality, 1D‐VNS provide unique biophysical, biomechanical, and biochemical cues when interfacing with live cells; VNS‐featured platforms have been developed as safe tools for manipulating activities of many cell types, including immune cells.^[^ ^7^, ^9^, ^26^, ^64^, ^65^ ^]^ The most prominently observed phenomena induced by 1D‐VNS at cellular level are nuclear deformation, plasma membrane remodeling, and cytoskeleton reorganization.^[^ ^7^, ^65^, ^66^ ^]^

#### 2.2.1 VNS‐Induced Nuclear Deformation

**Figure 3:**
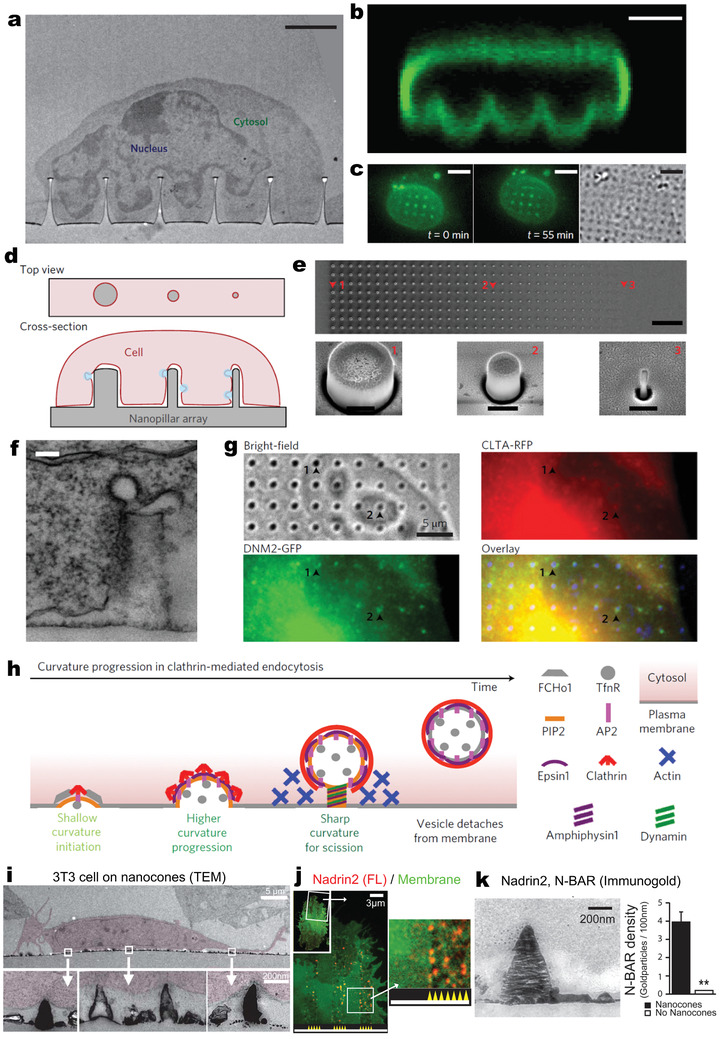
VNS‐induced cellular membrane deformations and endocytic processes. a) Transmission electron microscopy (TEM) image showing a cross‐section of an NIH‐3T3 fibroblast on nanopillars, with the nucleus deformed around nanopillars. Scale bar: 2 µm. b) Nuclear deformation visualized by immunostaining of Lamin A (green). Scale bar: 5 µm. c) Fluorescence (left and middle) and differential interference contrast (DIC, right) images of a live 3T3 cell transfected with GFP‐Sun2, demonstrating the nuclear deformation. Scale bars: 5 µm. a–c) Reproduced with permission.^[^ ^7^ ^]^ Copyright 2015, Springer Nature. d) Schematic of nanopillars with different radii (gray) deforming the cell membrane to generate different membrane curvatures (red line). e) Top: SEM image of a gradient nanopillar array with height 700 nm, pitch 3 µm, and radii ranging from 500 to 50 nm (left to right). Scale bar: 10 µm; Bottom: zoomed‐in SEM images of individual nanopillars as indicated in the top image. Scale bars: 400 nm. f) TEM image showing a clathrin‐coated pit at the membrane around a nanopillar. Scale bar: 100 nm. g) Immunostaining showing the accumulation of clathrin (CLTA‐RFP, red) and dynamin2 (DNM2‐GFP, green) at nanopillar locations. Scale bar: 5 µm. Arrowheads indicate locations of nanopillars with different radii. h) Schematic of proteins involved in different stages of clathrin‐mediated endocytosis (CME). d–h) Reproduced with permission.^[^ ^65^ ^]^ Copyright 2017, Springer Nature. i) TEM images of 3T3 cells grown on 200 nm nanocones. Cells are colored in red. Scale bars: 5 µm (top) and 200 nm (bottom). j) Confocal imaging of a 3T3 cell grown on nanocones and transfected with Nadrin2 (red) together with a membrane marker CAAX (green). k) Left, TEM image of an immunogold‐stained 3T3 cell transfected with fluorescently tagged N‐BAR domain of Nadrin2; right, statistical analysis of immunogold density measured over nanocones where membrane deformation was observed, compared with adjacent regions (no nanocones) within the same image. i–k) Reproduced with permission.^[^ ^64^ ^]^ Copyright 2012, Springer Nature.

The mechanical stability and deformability of the cell nucleus are crucial to diverse biological processes, including migration, proliferation, and polarization.^[^ ^67^, ^68^, ^69^ ^]^ For example, mechanical forces applied to nuclei can alter epigenetic regulation networks and modulate gene expression, ultimately affecting cellular functions.^[^ ^70^ ^]^ Neutrophil nuclear morphology has historically been used in hematology for neutrophil identification and characterization.^[^ ^71^ ^]^ Nuclei are the key organelle for genomic information storage and transcription, which are tightly regulated by 3D structure and mechanical features of chromatins.^[^ ^72^ ^]^ In this context, 1D‐VNS were demonstrated as an effective approach for noninvasive subcellular perturbation of nuclei, the deformation of which can be well‐controlled by varying VNS geometry. Spacing and diameter of vertical nanopillar arrays had a strong effect on nuclear deformation, where a larger pillar pitch and/or a smaller pillar diameter drastically increased the nuclear deformation, whereas the height of pillars had only a mild effect (**Figure** **3**a–c).^[^ ^7^ ^]^ By identifying how nuclei respond to topography and inherent mechanics, it is possible to program nanopatterned PDMS elastomers for directional nuclear deformation. VNS platforms can also be developed to characterize early changes in nuclear mechanics and morphology associated with a number of diseases, such as Hutchinson–Gilford progeria syndrome—a premature aging disease of children where abnormal nuclear shapes can be detected, including lobulation of the nuclear envelope, thickening of the nuclear lamina, loss of peripheral heterochromatin, and clustering of nuclear pores.^[^ ^73^ ^]^

#### 2.2.2 VNS‐Induced Membrane Remodeling and Cytoskeleton Reorganization

Aside from nuclear deformation, most engineered 1D‐VNS materials interface extensively with the cell membrane, which features heterogeneous but regulated structures and composition. Membrane heterogeneity plays an indispensable role in multiple cell functions, such as adhesion, migration, viability, and receptor‐mediated activation.^[^ ^74^ ^]^ In response to mechanical stimuli, immune cells can reorganize their cytoskeleton quickly—as fast as in few minutes.^[^ ^75^ ^]^ The mechanosensitive ion channels on immune cells’ plasma membrane can be opened through membrane tension.^[^ ^76^ ^]^ And mechanical force plays an important role in directly activating transmembrane receptors, such as B cell receptors (BCRs).^[^ ^77^ ^]^

Mesoporous Si nanoneedle arrays have demonstrated the ability to interact with multiple mechanoresponsive components simultaneously, inducing the reorganization of the lipid bilayer and membrane‐associated proteins, as well as the actin cytoskeleton beneath the cell cortex.^[^ ^78^ ^]^ A gradient array of SiO_2_ nanopillars with radii of 50–500 nm was fabricated to investigate the effect of pillar radius on membrane curvature (Figure 3d,e).^[^ ^65^ ^]^ TEM images showed that nanopillars could induce plasma membrane invaginations with sizes close to those of clathrin‐coated endocytic pits (Figure 3f). In addition, immunostaining results confirmed the local accumulation of two key proteins for clathrin‐mediated endocytosis—clathrin (CTLA) and dynamin2 (DNM2)—on highly curved membranes along pillars with radii <200 nm (Figure 3g); such curvature progression induced by nanopillars resulted in enhanced endocytosis (Figure 3h).

Tin oxide nanocones fabricated on a planar substrate also caused local inward membrane curvature (Figure 3i) and recruitment of N‐BAR domain proteins—versatile membrane‐associated regulatory elements that function over a wide range of cellular processes including regulation of cortical actin structures and endocytosis.^[^ ^64^ ^]^ Confocal imaging revealed that nanocones induced the selective accumulation of full‐length and isolated N‐BAR domains of Nadrin2, a regulator of actin polymerization (Figure 3j). TEM imaging confirmed that N‐BAR domains of Nadrin2 were recruited to the curved membrane around the nanocones (Figure 3k). This nanocone‐induced recruitment was also applied to the isolated N‐BAR domain of Amphiphysin1, a regulator of clathrin‐mediated endocytosis. Since endocytosis is a crucial initial step for immune cells to sense and engulf pathogens, leading to cascades of innate and adaptive immune responses, manipulating membrane curvatures and endocytic activation by 1D‐VNS are promising approaches for immune modulation.

#### 2.2.3 Prospects of 1D‐VNS in Probing Subcellular Activities

Aside from these 1D‐VNS tools for immune‐cell modulation, custom‐tailored cellular modification and subcellular‐level precision control are promising future directions in research and clinical study. Increasingly diverse nanotopographies have emerged in recent years.^[^ ^79^, ^80^, ^81^, ^82^ ^]^ Porous Si nanoneedles have shown the potential to be harnessed for cellular modulation at the organelle level, since cellular compartments such as cell membrane, cytoskeleton, and nucleus generated distinct responses to topographical cues.^[^ ^78^ ^]^ By electrochemical oxidation/reduction, a vertical polypyrrole (Ppy) array was reversibly switchable between nanotubes (highly adhesive hydrophobic) and nanotips (poorly adhesive hydrophilic).^[^ ^83^ ^]^ Such Ppy arrays provided dynamic stimuli that can alter surface adhesion, enabling controlled differentiation of mesenchymal stem cells at the nanoscale. Despite their promising potential, these newly developed VNS‐enhanced tools have not yet been applied to probe and regulate immune cell functions.

### 2.3 In Vitro Activation of Immune Cells by 1D‐VNS

Immune cells actively sense and respond to physical deformation and mechanical stress via mechanosensitive channels and the cytoskeleton, resulting in activation of immune pathways such as inflammasomes.^[^ ^76^ ^]^ So in addition to their fundamental role as research tools for understanding the immune system, 1D‐VNS platforms can directly modulate immune responses.

#### 2.3.1 Morphological Changes Induced by Nanopatterned Structures

**Figure 4:**
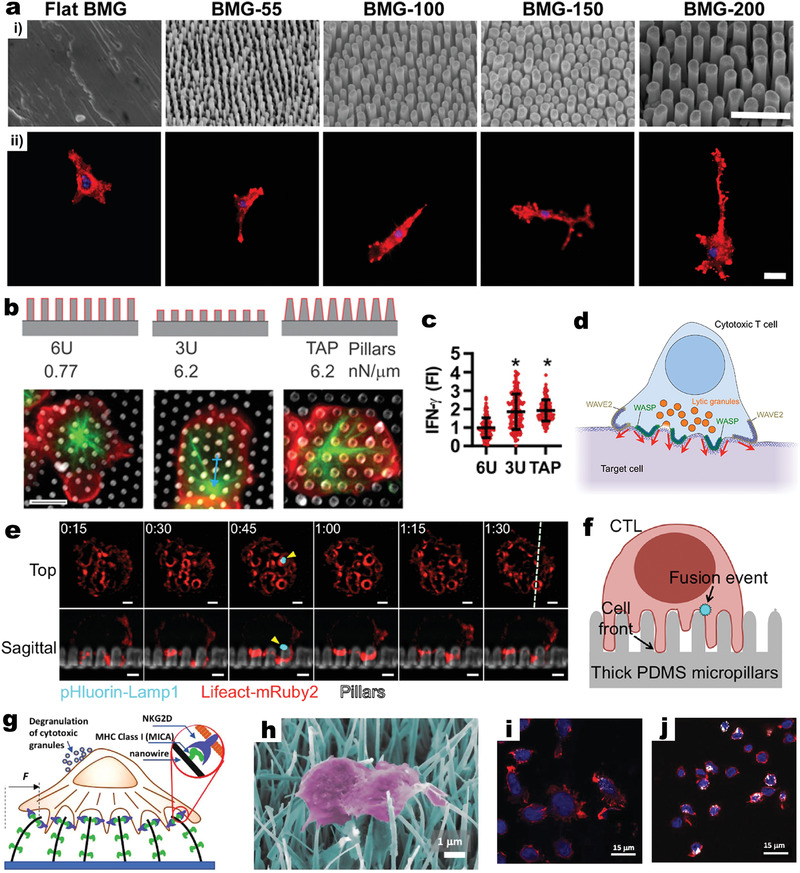
In vitro activation of immune cells by 1D‐VNS. a) SEM (i) and fluorescence (ii) microscopy images of morphological changes of macrophages induced by different nanopatterned (diameter ranging from 55 to 200 nm) BMGs. Scale bars: 1 µm (i) and 20 µm (ii). a) Reproduced with permission.^[^ ^9^ ^]^ Copyright 2014, ACS. b) Top: schematic of three PDMS pillar arrays used to test the effect of 3D structure and stiffness on T cell activation. Bottom: fluorescence images illustrating microtubule structure (green, β‐tubulin) and cell morphology (red, CD45) for cells on PDMS pillars. Scale bar: 5 µm. c) Statistical analysis demonstrating increased IFN‐γ secretion with increasing spring constant. *P < 0.001. b,c) Reproduced with permission.^[^ ^26^ ^]^ Copyright 2019, The Authors, published by National Academy of Sciences, USA. d) Schematic of the cytolytic immune synapse involving peripheral WAVE2‐dependent protrusions and central WASP‐dependent protrusions. The red arrows denote force exertion. e) Time‐lapse montage (image collected every 15 s) of a representative CTL cell expressing Lifeact‐mRuby2 (red) and pHluorin‐Lamp1 (bright blue) on PDMS pillars (gray). Z‐projection images (top views) are shown above with sagittal views below. The white dashed line (at 1 min 30 s, 1:30) denotes the slicing plane used for the sagittal images. Yellow arrowheads indicate the fusion event. Scale bars: 2 µm. f) Schematic of lytic granule fusion (visualized by pHluorin‐Lamp1 in (e)) on PDMS pillar arrays. d–f) Reproduced with permission.^[^ ^33^ ^]^ Copyright 2019, The Authors, published by AAAS. g,h) Schematic (g) and false‐colored SEM (h) of the activation of NK cells by MICA‐functionalized ZnO NWs through surface receptor NKG2D engagement. i,j) Fluorescence microscopy images showing NK cells on MICA‐functionalized flat control (i) and NWs (j). CD107a staining (white) indicates enhanced degranulation in NK cells cultured on NWs (j) compared with that on flat control (i). g–j) Reproduced with permission.^[^ ^29^ ^]^ Copyright 2018, Wiley‐VCH.

Arrays made of bulk metallic glasses (BMGs, diameter 55–200 nm; **Figure** **4**a,i) induced a change in the size, spreading, and elongation of various cell types.^[^ ^9^ ^]^ Fibroblasts decreased in cell area as the BMG diameter increased, and responded to BMGs of diameters as small as 55 nm. Endothelial cells detected BMGs of diameter 100 nm, but decreased significantly in cell size and elongation with larger BMG sizes. Unlike fibroblasts and endothelial cells, macrophages failed to detect BMGs of diameter ≤150 nm, but responded to those of diameter 200 nm, presenting more enlarged and elongated cell morphology (Figure 4a,ii).

Aside from influencing macrophage morphology, there is evidence that surfaces and scaffolds with nanotopographies can alter macrophage polarization between a proinflammatory M1 phenotype and an anti‐inflammatory/prohealing M2 phenotype.^[^ ^25^ ^]^ For example, a surface with nanopatterned grooves of 400–500 nm can drive murine bone‐marrow‐derived macrophages (BMMs) toward the M2 phenotype, secreting significantly higher levels of the anti‐inflammatory cytokine IL‐10, compared with BMMs cultured on a flat surface.^[^ ^24^ ^]^ This suggests that topography at the nanoscale can be designed to manipulate immune cell differentiation; but whether this and other types of advanced cellular manipulation can be applied to 1D‐VNS topography rather than groove structures is still to be determined.

#### 2.3.2 Enhanced Immune Responses via 1D‐VNS Perturbation

VNS can augment the induction of immune responses by enhancing a site‐specific endocytosis, a key process for APCs to effectively engulf extracellular antigens or dead tumor cells. CD4^+^ T cells formed complex interactions with elastomer PDMS pillar arrays; the dimension and flexibility of these anti‐CD3/anti‐CD28 antibodies‐coated pillars can affect CD4^+^ T cell activation. In particular, stiffer pillars (3 µm high, 3U, and tapered, TAP) with a larger spring constant (6.2 nN µm^−1^) delayed transport of the centrosome/microtubule‐organizing center (MTOC) toward the middle of the cell–pillar interface (Figure 4b)—a key step in T cell activation and immunological synapse (IS) formation. Stiffer pillars also promoted significantly higher expression level of IFN‐γ in CD4^+^ T cells, compared with that using less stiff (6 µm high, 6U; 0.77 nN µm^−1^) ones (Figure 4c).^[^ ^26^ ^]^ These results reveal a complex effect of VNS‐substrate mechanics on cellular responses from MTOC centralization to cytokine secretion.

Similarly shaped PDMS pillars were used to interact with CD8^+^ CTL cells. These pillars induced the deformation of CTL plasma membrane, stimulating formation of actin‐rich protrusions, assisted by two major nucleation‐promoting factors–WASP and WAVE‐2 (Figure 4d). These protrusions were necessary for synaptic force exertion, particularly in more central regions of the IS close to lytic granules; they were also required for physical deformation of target cells in bona fide cytolytic interactions. It was deduced that the engineered interface between CTL cells and PDMS pillars resembled the growth of communicative IS between CTLs and target cells in 3D. This unique approach helped identify the key role of actin protrusions in promoting synaptic force exertion and CTL killing, with elevated degranulation of granzymes and perforins (cytotoxic proteins injected into target cells via IS by CTLs). Granule fusion events were detected in single‐cell imaging experiments, using a fluorescent reporter containing a pH‐sensitive GFP (pHluorin) fused to the granule‐targeting domain of Lamp1 (pHluorin‐Lamp1); F‐actin protrusion were visualized via Lifeact‐mRuby2 or by staining with a fluorescent Fab against the surface marker CD45. Time‐lapse observations of a 1 µm diameter synaptic domain demonstrated that F‐actin accumulation increased modestly within this domain during granule fusion, indicating that cytolytic secretion and protrusion growth occurred concurrently in the same region (Figure 4e, top). Linescans of sagittal slice images revealed that F‐actin did not overlap with the fusion site but accumulate underneath it (Figure 4e, bottom), implying that granule fusion occurred in F‐actin‐free zones that formed transiently at the base of active F‐actin‐rich protrusions on PDMS pillars (Figure 4f).^[^ ^33^ ^]^

With adjustable spatial and mechanical properties, such vertically aligned elastomer micro/nanostructures give advantages over planar substrates for their ability to capture the complexity of immune cellular network at play under environment close to real physiological conditions.

#### 2.3.3 Role of VNS Surface Functionality in Modulating Immune Cell Function

Surface chemistry also plays an important role in immune cell stimulation. Controlled surface nanotopography by gold nanoparticle immobilization combined with carboxyl acid rich coatings enhanced the secretion of matrix metalloproteinase‐9 (MMP‐9)—an enzyme involved in tissue remodeling and foreign body responses—by murine primary neutrophils, without significant impact on other markers of neutrophil functionality.^[^ ^23^ ^]^ In regards to BMMs, nanotopography modified with carboxyl acid functionality significantly impaired the release of inflammatory cytokines (including IL‐6 and IL‐1β) after LPS stimulation; but other surface modification, such as amine or methyl group modification, showed less pronounced effects.

In addition to simply modifying VNS surfaces with generic polymers to obtain different charges or hydrophilic properties, coating with receptors and/or ligands to activate particular target cells could further strengthen the capability of VNS‐mediated immune modulation. For instance, surface functionalization of ZnO NW arrays with a specific antigen—major histocompatibility complex class I‐related chain A (MICA)—greatly enhanced the binding and activation of NK cells via the surface receptor NKG2D (Figure 4g,h).^[^ ^29^ ^]^ MICA ligands provide essential biochemical cues to evoke NK cells, since nonfunctionalized and mock‐functionalized NWs produced no significant NK cell activation. But once triggered, the effects of mechanical cues from NW topography overruled the effects of MICA quantity on NK immune response. MICA‐functionalized NWs stimulated four times the expression of CD107a (exposed to NKs’ outer membranes during activation/degranulation), compared with that on MICA‐coated flat substrates (Figure 4i,j). This is despite the fact that ≈30 times the amount of MICA was exposed to NK cells on the flat substrates compared with the NW array substrates, where only the tips (the top 10%) of NWs interacted with cells. Such surface‐functionalized NWs could enable study of immune responses under multiple stimuli that are more relevant to the in vivo milieu.

### 2.4 In Vivo Immunomodulation by VNS Topography

In vivo modulation of immune cells by nanotopography is an important direction, permitting rapid local regulation of immune responses with biophysical cues.

**Figure 5:**
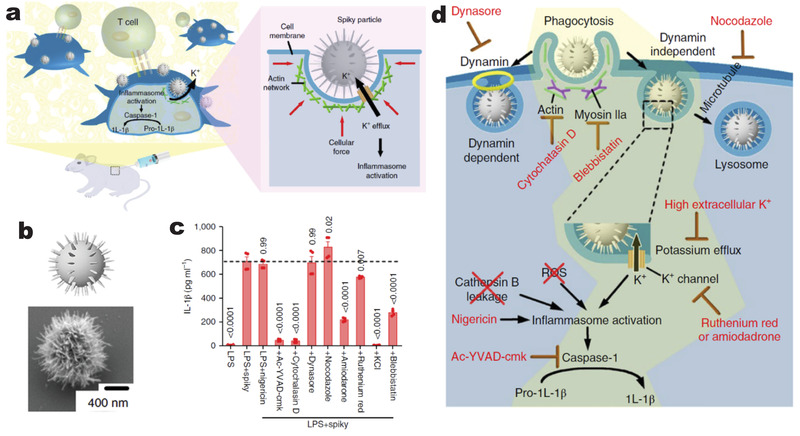
VNS‐induced immunomodulation in vivo. a) Schematic of spiky particles applied to activate immune cells and amplify immune responses in vivo. b) Schematic (top) and SEM image (bottom) of a spiky particle. c) Statistical analysis of inflammasome activation in BMMs that were pretreated with indicated inhibitors for 0.5 h and treated with LPS and spiky particles. The dotted line indicates the mean of the LPS^+^ spiky group. d) Schematic of possible inflammasome activation mechanisms stimulated by spiky particles. The yellow region indicates a feasible mechanism supported by the experimental results. a–d) Reproduced with permission.^[^ ^34^ ^]^ Copyright 2018, Springer Nature.

Most studies of VNS‐mediated immunomodulation discussed above are based on planar substrates. Notably, a distinct strategy was developed to grow a corona of nanospikes on TiO_2_ microparticle surface, designated as “spiky particles,” to stimulate innate immune responses in vivo (**Figure** **5**a,b).^[^ ^34^ ^]^ The spiky nanotopography on microparticle surfaces promoted activation of inflammasomes (cytosolic multiprotein oligomers responsible for the activation of inflammatory responses during innate immunity) in both BMMs and DCs. Further study demonstrated that spiky particles introduced mechanical stress on the cell membrane, leading to the activation of mechanosensitve potassium channels to allow K^+^ efflux, which is likely to be driven by myosin IIa—a key contractile motor protein that generates mechanical forces during phagocytosis. Spiky particles also provoked downstream IL‐1β release in a caspase‐1‐ and NLRP3‐dependent fashion (Figure 5c). Inhibition of K^+^ efflux with a high extracellular K^+^ concentration completely abrogated the spiky‐particle‐induced inflammasome activation; while treatment with K^+^ channel inhibitors, amiodarone or ruthenium red, significantly blunted the release of IL‐1β from BMMs treated with spiky particles (Figure 5d).

Spiky particles coupled with monophosphoryl lipid A (MPL)—an agonist of TLR4—effectively activated tumor antigen‐pulsed DCs, which was subsequently used as a cancer DC vaccine to eliminated murine tumors. When injected together with MPL, spiky particles promoted DC maturation in vivo, and robustly augmented vaccine‐induced adaptive immunity against influenza viral infection.^[^ ^34^ ^]^ Importantly, in vivo activation of immune cells by nanotopography suggests a new strategy in addition to conventional biochemical stimulation, creating a basis for engineering more potent vaccines and adjuvants for immune system activation. Given its potent adjuvant effects over conventional chemical adjuvants, a combination of spiky particles and immune stimulators could be used for “in situ” cancer vaccines that have already shown initial success in multiple tumor types.^[^ ^84^, ^85^ ^]^ Intratumoral injection of this combination after local ablation of tumors by radiation or radio frequency ablation treatment may induce robust tumor‐specific immune responses and prohibit recurrence or metastasis.^[^ ^86^, ^87^, ^88^ ^]^

The demonstration of spiky particles to activate immune response has confirmed the applicability of VNS‐mediated immunomodulation in vivo. But current spiky particles are made of nondegradable materials, which limit application in prophylactic vaccines for healthy populations. The development of biodegradable spiky particles could fully realize the potential of 3D‐structured VNS for in vivo immune modulation in vaccination and cancer immunotherapies.

### 2.5 Discussion of Immunomodulation by VNS Topography

These elegant studies are early evidence that 1D‐VNS could modulate activities of various cell types by inducing cellular deformation and membrane curvature, mediating reorganization of cytoskeleton and opening of mechanosensitive channels. Because immune cells are sensitive to mechanical forces, it is highly likely that 1D‐VNS devices have extensive immune modulation effects, not limited to cell types and immune processes so far reported. But current VNS studies have worked on a finite number of immune cell types, omitting key players and their subpopulations in the induction of an immune response; one of these missing players is the B cell.

B cells are an important immune cell type likely to respond to mechanical stimuli via BCR.^[^ ^89^, ^90^ ^]^ B cell activation requires physical interaction with professional APCs—follicular DCs (FDCs)—which display protein fragments derived from infecting pathogens on their surface.^[^ ^91^ ^]^ BCRs recognize these fragments and assemble into microclusters; signaling proteins and adaptors accumulate at developing microclusters, which trigger transduction pathways, initiating transcription and cell activation.^[^ ^90^ ^]^ Ligand mobility can modulate BCR diffusion and clustering, which are important in early B cell signaling.^[^ ^92^, ^93^ ^]^ DNA‐based nanosensors were developed to examine the role of mechanical cues from IS in regulating B cell responses.^[^ ^94^ ^]^ The results showed that B cells primarily used mechanical forces to extract antigens from IS, and resorted to enzymatic liberation only if mechanical forces failed to do so. The same study also found that the stiffer FDCs promoted strong B cell pulling forces and stringent affinity discrimination, whereas the softer DCs promoted extraction of low‐affinity antigens by weak pulling forces; this suggested that distinct physical properties of APCs supported different stages of B cell activation. B cells have now been exploited as promising targets for nanoparticle‐based vaccines against viral and bacterial infections, due to their essential role in humoral and memory immune responses.^[^ ^95^, ^96^, ^97^ ^]^ However, less work has been devoted to exploring the use of 1D‐VNS platforms in generating mechanical forces and biophysical cues for BCR activation and B cell signaling. It would be interesting to investigate how B cells behave on an antigen‐conjugated VNS of different size, geometry, and rigidity; and how such VNS‐interfacing alters B cell activation, maturation, and antibody production during humoral responses—all of which would guide innovative treatment and vaccination strategies.

Despite evidence for VNS‐mediated immunomodulation effects, the underlying molecular mechanisms require further investigation, including but not limited to the identification of primary mechanical sensors and subsequent signaling transduction cascades. A computational model suggests that local membrane curvatures—which can be induced by well‐designed VNS—lead to the opening of mechanical sensing channels,^[^ ^98^ ^]^ which play an evolutionary role from fungi to mammals in sensing environmental mechanic stress.^[^ ^75^, ^99^ ^]^ Deformation and rearrangement of cell cytoskeletons in response to mechanical forces induced by VNS were shown to activate several cellular signaling pathways, such as inflammasomes.^[^ ^75^, ^100^ ^]^ A recent study produced insights into how membrane curvature generated by VNS was transduced into aforementioned biological processes.^[^ ^101^ ^]^ A F‐BAR domain containing protein FBP17 was identified to be a key curvature‐sensing protein. It was found that FBP17 could activate downstream signaling pathways to nucleate formation of branched F‐actin; this subsequently affected a number of cellular processes, including endocytosis, stress fiber organization, and focal adhesion maturation.

The interactions between immune cells and 1D‐VNS are complex and dynamic. Taking the initial progress described above as a start, a more systematic study involving a comprehensive comparison among shapes, sizes, geometry, rigidity, and chemical modification are likely to yield important advances. There are now significant opportunities for extending knowledge of how precisely engineered 1D‐VNS can manipulate immune responses, both ex vivo and in vivo. Further exploration of the underlying mechanisms will have profound implications for VNS‐based multifunctional metamaterials in early diagnosis and synergistic cancer immunotherapy, such as ACT and cancer vaccines. VNS‐based ex vivo activation of DCs could serve as a platform to optimally expand tumor‐specific T cells that are infused back into the patient to eliminate tumor cells. On the other hand, injectable 1D‐VNS such as spiky microparticles, can not only drive ex vivo activation of immune cells, but modulate immune responses in vivo when injected into the body, serving as an effective cancer vaccine adjuvant.

## 3 VNS‐Mediated Intracellular Delivery Into Immune Cells

### 3.1 Overview of Current Intracellular Delivery Methods

Intracellular delivery of genetic materials into living cells regulates the transcription and translation of exogenous genes into specialized proteins using a cell's own machinery. This valuable paradigm has been widely used in molecular biology and biomedical research, such as ex vivo immuno‐oncology, T cell reprogramming, and genome editing. In the burgeoning field of cell‐based immunotherapy, CAR‐T therapy as an example, the CAR gene (in the form of DNA or mRNA) is delivered into human T cells, eliciting specific anti‐tumor immunity.^[^ ^102^ ^]^ Current delivery mechanisms can be divided into two broad categories: viral transduction and nonviral transfection (including chemical and physical methods).

#### 3.1.1 Viral Transfection

The process of using a nonreplicating viral vector to deliver foreign DNA into a cell is called transduction. Primary cells such as human‐induced pluripotent stem cells (iPSCs) and immune cells are notoriously hard to transfect. This is particularly true for lymphocytes, which have a thinner cell membrane with lower protein content, making them more susceptible to cell death induced by electroporation and transfection agents than adherent cells.^[^ ^103^, ^104^, ^105^ ^]^ In addition, without antigen stimulation, materials delivered are less likely to localize into the nucleus of a nondividing lymphocyte than into that of a dividing one. So viruses, especially lentivirus, are commonly used to transfect immune cells, due to their innate ability to transduce nondividing/resting cells. Viral transduction is robust, highly efficient, and can lead to long‐term expression; but it has drawbacks such as a costly and lengthy procedure, restricted cargo packaging, the risk of insertional mutagenesis, and undesirable T cell activation—all serious hurdles to developing cell‐based therapies.

#### 3.1.2 Chemical Transfection

Chemical transfection is a popular technique due to the ease, cost, and wide variety of transfection reagents available for purchase. For example, lipofection using cationic liposomes (lipid‐based or nonlipid‐based) is commonly used for short‐term expression of a desired gene that lasts a few days. Lipofection can achieve high transfection efficiency in immortalized cells, but have low efficiency on primary lymphocytes.^[^ ^106^ ^]^ In particular, T cells have shown resistance to common lipofection reagents; this might be due to their low expression of exostosin‐1, a key enzyme in the biosynthesis of heparan sulfate (HS) proteoglycans. Since HS proteoglycans donate a negative charge to the cell surface, the lack of HS on lymphocytes results in a relatively high positive surface charge; this might increase the sensitivity of lymphocytes to cell lysis by cationic vectors due to excess local positive charge. It has also been shown that transfection of lymphocytes using conventional cationic reagents can lead to apoptosis, necrosis, and secretion of TNF‐α.^[^ ^107^, ^108^ ^]^

#### 3.1.3 Physical Transfection

Numerous physical intracellular delivery methods have been developed to deliver a range of bioactive cargoes into a broad range of cells, all of which are to some extant capable of in vitro delivery efficiency. Among these, microinjection and bulk electroporation (BEP) are the most common, and have been used for effective DNA transfection in range of suspension cells such as primary lymphocytes. Microinjection is simple and can be very efficient when optimized, but it is a time‐consuming and laborious procedure; only one cell can be injected at a time, largely limiting the amount (only a few hundred) of cells to be transfected per experiment.^[^ ^109^ ^]^ In BEP, cells are suspended between two parallel electrodes and the cell membrane is artificially ruptured under an applied electric field of up to a few thousand volts, leading to adverse effects such as Joule heating, gas bubble formation, and unwanted electrochemical reactions and pH variations—all of which contribute to poor cell viability and inconsistent delivery outcomes.^[^ ^110^ ^]^ Critically, a recent report has shown that BEP can trigger an alteration in the expression of immune‐associated genes related to immune cell activation and trafficking.^[^ ^111^ ^]^

Due to the disadvantages of existing transduction/transfection methods, there is still a need for a safer nonviral and universal delivery technique that yields high transfection efficiency on primary lymphocytes; ideally, the technique should preserve cell viability and immunological competence which is critical for potential clinical intervention.

### 3.2 Early Success of Biomolecular Delivery via 1D‐VNS

Delivery by 1D‐VNS has shown great promise for overcoming the limitations of existing delivery methods; the unique topological morphologies of 1D‐VNS allow rapid and direct intracellular access of a wide range of bioactive cargos—such as DNAs, RNAs, proteins, and their complexes—with minimal impact on cell viability and function.^[^ ^6^, ^13^, ^15^, ^36^, ^112^, ^113^, ^114^, ^115^, ^116^, ^117^ ^]^

**Figure 6:**
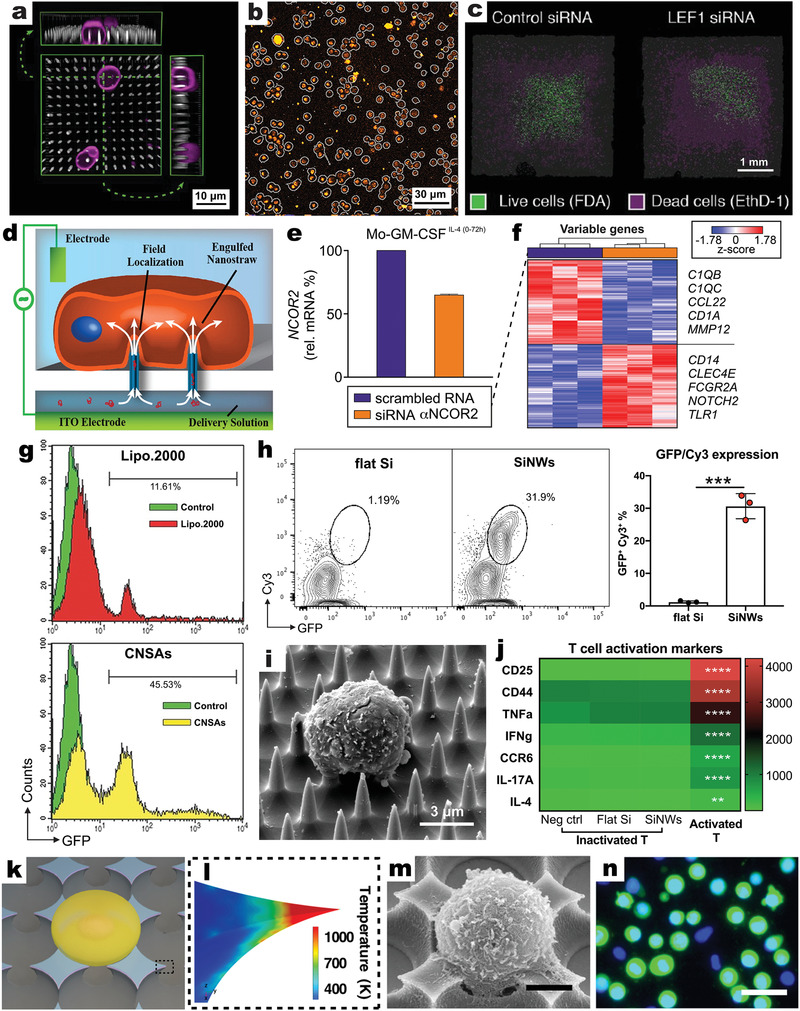
VNS platforms facilitate efficient transfection of nucleic acids into primary immune cells. a) 3D reconstruction of confocal microscopy imaging showing the interface between SiNWs (white) and human B cells (membrane: magenta). b) Confocal microscopy image showing SiNW‐based delivery of Cy3‐siRNA (orange) to human B cells (intact cytoplasms: gray outlines). c) Confocal image of CLL‐B cells (intact cytoplasms: green, dead nuclei: magenta) on SiNW samples (dark gray squares) loaded with nontargeting siRNA (left) and anti*‐LEF1* siRNA (right). a–c) Reproduced with permission.^[^ ^28^ ^]^ Copyright 2012, ACS. d) Schematic of the field localization and biomolecule confinement at the tip of the nanostraws in NES. Reproduced with permission.^[^ ^122^ ^]^ 2013, ACS. e) Quantification of relative *NCOR2* mRNA level in monocytes transfected with control scramble RNA (purple) and anti‐*NCOR2* siRNAs (orange). f) Heatmap of the top 1000 genes being most variable induced by *NCOR2* silencing, with a few genes depicted on the right side to represent upregulated and downregulated genes. e–f) Reproduced under the terms of the CC‐BY Creative Commons Attribution 4.0 International license (https://creativecommons.org/licenses/by/4.0).^[^ ^120^ ^]^ Copyright 2017, The Authors, published by Elsevier. g) Histogram of the GFP expression within lymphocytes transfected with pEGFP plasmids using control group Lipo.2000 (top) and carbon nanosyringe arrays (CNSAs, bottom). Reproduced with permission.^[^ ^38^ ^]^ Copyright 2016, Wiley‐VCH. h) Flow cytometric and statistical analysis of Cy3^+^ GFP^+^ population of primary mouse T cells 48 h after harvesting from flat Si and SiNWs coated with Cy3‐tagged GFP plasmids. i) SEM image of the interface of a primary mouse T cell on an array of SiNWs. j) Heatmap of the expression of key activation markers within inactivated T cells cultured in well‐plate (Neg ctrl), on flat Si, and SiNWs, as well as activated T cells. h–j) Reproduced with permission.^[^ ^36^ ^]^ Copyright 2019, Wiley‐VCH. k) Schematic of a cell in a microwell. l) Finite element method simulation showing the hot spots at a sharp nanotip as indicated in (k). m) SEM image of a Ramos B cell inside a microwell. n) Fluorescence image of Hoechst 33342 stained (blue) cells on microwells preloaded with 4 kDa FITC‐dextran (green). Scale bars: 3 µm (m) and 40 µm (n). k–n) Reproduced with permission.^[^ ^123^ ^]^ Copyright 2019, ACS.

NWs with controlled geometry have achieved high siRNA transfection efficiency (>95%) for different types of primary immune cells, including adherent (DC, macrophage, NK) and nonadherent (T and B, **Figure** **6**a,b) immune cells, with minimal immune response activation.^[^ ^28^ ^]^ For instance, SiNW‐facilitated transfection of siRNAs that silence the expression of *LEF1* gene—a terminal transcriptional activator and a canonical target of the Wnt signaling pathway—severely impaired the survival of human B cells obtained from a chronic lymphocytic leukemia (CLL) patient (Figure 6c, right). At the cellular level, the Wnt signaling pathway contains more than 100 members and is critical for the proliferation and cell fate determination of many cell types, including B cells.

In a follow‐up study, the same SiNW platform was used to deliver siRNAs into primary CLL‐B cells to assess the impact of gene knockdown on CLL survival.^[^ ^118^ ^]^ Based on the DNA sequencing results of a series of CLL samples, they interrogated the effects of 8 of 15 mutated Wnt pathway members identified across 91 CLLs. The research demonstrated that inhibition of the Wnt pathway at different levels adversely affected CLL survival. CLL samples harboring gain‐of‐function mutations (*DKK2*, *BCL9*, *RYK*) exhibited greater dependency on Wnt pathway signaling. Silencing these mutated genes in CLL‐B cells resulted in reduced viability compared with wild‐type B cells. Decrease in CLL cell survival was also observed when silencing *CSNK1E*, the mutation of which led to loss of Wnt pathway activation.

Despite the readout on cell viability alone, the SiNW‐mediated gene delivery approach described in those two studies helped to gain insights into Wnt/β‐catenin regulatory molecular circuits and how mutated genes and pathways are involved in the pathogenesis of CLL. Genetic characterization of the Wnt signaling can identify subgroups of CLL patients with greater sensitivity to targeting of this pathway, affording a potentially more fruitful and less toxic approaches for effective therapeutic interventions. Furthermore, since SiNWs demonstrated highly efficient (>90%) delivery of fluorescently labeled siRNAs without compromising cellular viability (>95% survival), this approach overcame the poor efficiency of conventional transfection methodologies to genetically manipulate primary CLL‐B cells.^[^ ^118^ ^]^

In a mechanistic and seminal study, molecular circuits that control differentiation of naïve T cells were probed in order to understand how extra‐ and/or intracellular signals are processed into coherent cellular responses and determine cell fate.^[^ ^27^ ^]^ When combined with high‐resolution transcriptional profiling and novel computational algorithms, SiNW‐based perturbation tools were used for effective and functional interrogation of primary immune cells. SiNWs mediated efficient transfection of selected siRNAs into murine CD4^+^ T cells, leading to the identification of 39 regulatory genes (12 of them novel) and two mutually antagonistic regulatory circuits—all involved in the differentiation of naïve CD4^+^ T into Th17 cells. Crucially, SiNW‐mediated delivery did not activate immune responses or interfere with normal immune sensing mechanisms. This approach allowed pertubation of a molecular circuit's output (such as DNA replication and mRNA expression) via exogenous biomolecules (such as siRNAs and therapeutic materials) delivered via SiNWs.^[^ ^27^ ^]^ So by compartmentalizing a SiNW array, it is feasible to realize high‐throughput gene silencing by delivering different siRNAs into B cells at designated areas on the array. This is particularly useful for studying how silencing specific genes affects B cell responses to a drug, which can then be used to predict therapeutic outcomes for CLL patients with specific genetic mutations.^[^ ^119^ ^]^

A separate study used SiNWs to administer a small‐molecule inhibitor of the Plk enzyme into BMDCs, demonstrating the essential role of Plk2 and Plk4 in regulating antiviral gene expression.^[^ ^37^ ^]^ Using a nanostraw‐electroporation system (NES) (Figure 6d), anti‐*NCOR2* siRNAs were introduced into human monocytes, reducing *NCOR2* mRNA levels to 65% relative to the control that were transfected with scrambled RNAs (Figure 6e). *NCOR2* silencing resulted in the enrichment of genes that were found downregulated in monocytes treated with IL‐4; this indicated that *NCOR2* is a key regulator for IL‐4‐induced monocyte differentiation (Figure 6f).^[^ ^120^ ^]^

VNS substrates can mediate delivery of larger and more complex macromolecules, such as plasmid DNAs containing therapeutic genes, to modify immune cells; this is a considerable advantage for both basic and translational biomedical research. For example, CNSAs achieved significantly higher transfection efficiency (≈46%) of pEGFP plasmids into primary lymphocytes than control Lipofectamine 2000 (Lipo.2000) (≈12%) (Figure 6g).^[^ ^38^ ^]^ In a more recent study, primary mouse T cells—in an unstimulated status—were successfully transfected (>30% efficiency) with Cy3‐tagged GFP plasmids coated on SiNWs, under a forcible interfacing by applied centrifugation (Figure 6h–j). Transfected T cells expressed GFP within 48 h after detachment from SiNWs. Of note, NW‐treated T cells maintained high viability and proliferative capacity, with no discernible increase in key activation markers (Figure 6j);^[^ ^36^ ^]^ this overcame significant limitations of conventional viral and nonviral methods that may induce unexpected immunogenicity and altered cellular function.^[^ ^121^ ^]^

Besides classic vertically aligned NW arrays, unconventional VNS‐featured platforms have also been developed to fit suspension immune cells. Specifically, a photothermal delivery platform was generated to deliver a range of extracellular cargoes into Ramos B cells.^[^ ^123^ ^]^ This platform was based on a uniform microwell array with 3D metallic sharp nanoscale tips (nanotips) at the edges of microwells (Figure 6k), which served as integrated local hot spots (Figure 6l) upon laser irradiation, and provided controllable transient membrane disruption for each cell in the array. Ramos B cells settled by gravity within each microwell in direct contact with eight sharp nanotips (Figure 6m). Subsequent laser treatment induced cavitation bubbles (phenomenon in which rapid changes of pressure in a liquid lead to the formation of small vapor‐filled cavities) and created transient pores in the cell membrane, permitting efficient transfection of different‐sized cargoes, with >84% for Calcein green (0.6 kDa), >45% for FITC‐dextran (2000 kDa), and >58% for GFP‐reporter plasmids. The bacterial enzyme β‐lactamase (29 kDa) was also delivered into Ramos B cells while retaining its biological activity. Importantly, cells maintained high viability (>90%) after transfection. The study suggests that a microwell–nanotip integrated platform can manage the number, location, and size of poration on suspension cells to achieve high transfection efficiency and high cell viability, if there is close control over experimental parameters such as cavitation bubble size (using different laser fluences), and pore number (using juxtaposed metallic nanotip configurations).

The specific 3D topography of VNS not only provides a large surface area and a high local density of ligands presentation, but enhances local intimate and dynamic interactions between the VNS surface and cellular components at the nanoscale.^[^ ^124^ ^]^ But unlike adherent cells that anchor naturally and extensively on many VNS substrates, the suspension lymphocyte cells are only in loose contact with most VNS and can easily detach with subtle external perturbations. Therefore, the cell–VNS interface needs to be well‐configured to stabilize and enhance the adhesion of suspension cells for efficient cargo delivery.

### 3.3 Integration of Synergistic Routes to Maximize VNS‐Mediated Delivery

Research on developing smart and functional mechano‐instructive surfaces for VNS‐mediated transfection technology is now attempting to balance multiple independent parameters and their complex interactions at play in a cell culture environment (VNS topography and patterning, surface functionality and presentation, and cell and cargo types),^[^ ^28^, ^36^, ^125^, ^126^ ^]^ with combinatorial intracellular delivery approaches such as applied external force,^[^ ^36^, ^127^, ^128^ ^]^ cell squeezing,^[^ ^129^ ^]^ electroporation,^[^ ^112^, ^122^, ^130^ ^]^ oscillation,^[^ ^14^, ^131^ ^]^ and laser irradiation.^[^ ^132^, ^133^ ^]^ Understanding this balance has the potential to maximize VNS transfection efficiency by generating multifunctional integrated systems, which can facilitate cell attachment/detachment, strengthen cell–VNS interface, enhance membrane permeability, activate cellular uptake processes, and control biomolecular release.

#### 3.3.1 Enhanced Cell–VNS Interface by Surface Functionalization and Applied External Force

**Figure 7:**
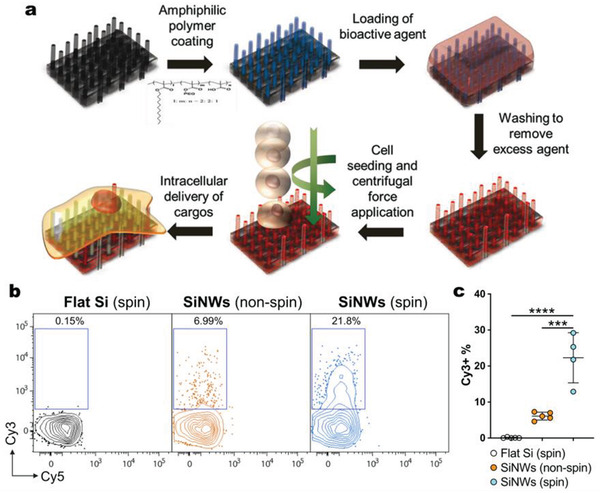
External active force by centrifugation enhances VNS‐mediated transfection. a) CNSA‐mediated intracellular cargo delivery under applied centrifugation. Reproduced with permission.^[^ ^38^ ^]^ Copyright 2016, Wiley‐VCH. b,c) Flow cytometry analysis (b) and quantification (c) of Cy3^+^ population of L1.2 cells harvested after 6 h incubation on flat Si (spin), SiNWs (nonspin), and SiNWs (with spin), coated with Cy3‐tagged plasmids (100 ng µL^−1^). b,c) Adapted with permission.^[^ ^36^ ^]^ Copyright 2019, Wiley‐VCH.

Selective VNS surface functionalization has shown the capacity for a broad biophysiochemical tunability, allowing a more efficient interfacing with biological systems at play as well as the ability to facilitate VNS internalization events to achieve specific and controlled transfection in immune cells. For example, an amphiphilic polymer consisting of polyethylene glycol and dodecyl alkyl units was used to coat CNSAs, making the surface hydrophilic so that aqueous cargoes could be loaded to the interior of individual carbon syringes (**Figure** **7**a).^[^ ^38^ ^]^ Cationic polymers such as poly‐d‐lysine, polydopamine, and polyornithine, and cell matrix noncollagenous proteins such as fibronectin and laminin were variously used for NW functionalization to enhance plasmid adsorption and promote cell adhesion via electrostatic interactions.^[^ ^36^, ^125^, ^134^, ^135^, ^136^ ^]^ Linker molecules such as 3‐aminopropyltrimethoxysilane and 3‐aminopropyltriethoxysilane were used to adsorb biomolecules (e.g., siRNAs, DNAs, and proteins) on the SiNW surface,^[^ ^126^, ^137^ ^]^ facilitating their delivery into naïve T cells without activating significant immune response.^[^ ^28^ ^]^ Stimuli‐responsive polymers grafted on VNS can modulate cell–VNS interactions by controlling cell‐capture and cargo‐release.^[^ ^138^, ^139^ ^]^ In particular, Si nanopillars and SiNWs modified with thermoresponsive poly(*N*‐isopropylacrylamide) were developed for temperature‐induced reversible capture and release of circulating tumor cells.^[^ ^140^, ^141^ ^]^ Using multiple levels of functionalization, gold nanopillar arrays were produced with spatio‐selective surface chemistry; the top of pillars was grafted with gold nanoparticles, whereas the bottom of pillars and the background were modified with a bioadhesive peptide.^[^ ^142^ ^]^ Bifunctionality of the gold nanopillar arrays creates a new paradigm for biological, biosensing, and biocatalysis applications.

In addition, there is now ample evidence that cell–VNS interactions enhanced via the application of external force, such as centrifugation^[^ ^16^, ^36^, ^38^ ^]^ (Figure 7a) and aspiration flow,^[^ ^143^ ^]^ can assist in intracellular access and delivery, sampling, and cellular interrogation.^[^ ^127^ ^]^ For example, it was reported that with a spin at 200 g for 15 min, the SiNW‐mediated delivery efficiency of Cy3‐tagged plasmids into suspension immune L1.2 cells was boosted by a factor of three compared with that without spin (nonspin) (Figure 7b,c).^[^ ^36^ ^]^

#### 3.3.2 Control of Cell “Capture‐and‐Release” on VNS Platforms

A recent study reported an “all‐in‐one” 1D‐VNS platform for optimal cellular engineering, including immune cells.^[^ ^143^ ^]^ Cell surface in the immune system is highly controlled and fine‐tuned through the complex mixture of carbohydrates structures (glycans).^[^ ^144^ ^]^ There is increasing evidence that suspension cells such as cancer and immune cells overexpress sialic acids (SAs). SAs motives typically conjugated to the termini of cellular glycans and play crucial role in fundamental cellular and molecular processes that regulate both stimulatory and inhibitory immune pathways.^[^ ^145^ ^]^

**Figure 8:**
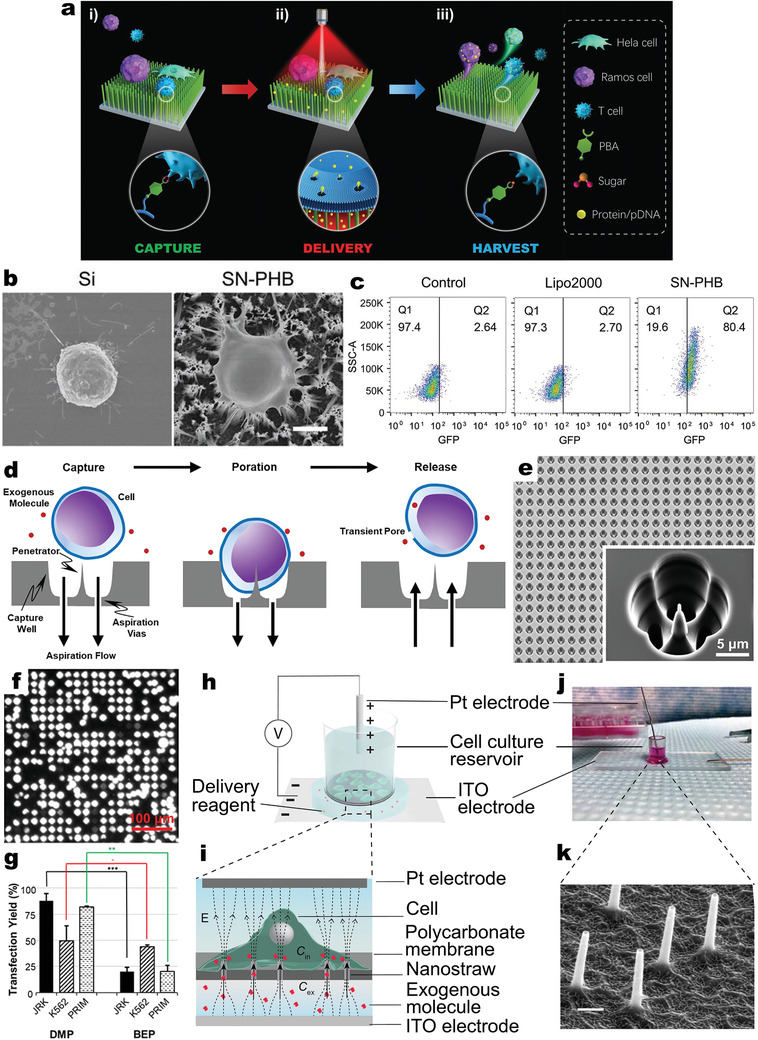
Synergistic routes used to maximize VNS‐mediated delivery. a) Schematic of the sugar‐responsive polymer (SN‐PHB) platform in capture‐delivery‐harvesting performance: i) Capture step: SiNW arrays were modified with an SN‐PHB containing PBA groups, recognized by glycoproteins and SAs on the cell membrane, thus promoting the capture of both adherent (HeLa) and suspension (Ramos and T) cells; ii) Delivery step: application of NIR induced the photothermal properties of SiNWs that facilitate efficient cargo (proteins/pDNAs) delivery through membrane disruption; iii) Harvest step: treatment with sugar solution broke the boronate ester bonds between PBA and glycoprotein/SAs, leading to the release of cells from SiNWs. b) SEM images showing Ramos cells on flat Si (left) and SN‐PHB surfaces (right). Scale bar: 5 µm. c) Flow cytometry detection of GFP expression in nontreated T cells (control, left), and T cells transfected with pGFP by Lipo.2000 (middle) and SN‐PHB system (right). a–c) Reproduced with permission.^[^ ^143^ ^]^ Copyright 2019, Wiley‐VCH. d) Schematic of the steps of DMP‐mediated cargo delivery: cells are captured using negative aspiration flow (left), porated by impingement upon the penetrator (middle), and released by reversal of flow after intracellular delivery (right). e) SEM image of a portion of DMP device (inset: higher magnification image of a single capture). f) Representative fluorescence microscopy imaging showing the occupancy of cell‐capture sites on DMP device array. g) Plots of transfection yield for DMP versus conventional BEP for Jurkat (JRK), K‐562 (K562), and primary human T cells (PRIM) (*p ≤ 0.05, **p ≤ 0.01, ***p ≤ 0.001). d–g) Reproduced with permission.^[^ ^149^ ^]^ Copyright 2020, ACS. h–k) Design and operation of the NES. h,j) Schematic (h) and photo image (j) of cells cultured on the NS membrane in a well plate; the delivery reagent is placed under the bottom of the reservoir and an electric field is applied between the platinum and ITO electrode. i) Schematic of NES delivery mechanism. k) SEM image of the NS array on the membrane. Scale bar: 300 nm. h–k) Reproduced with permission.^[^ ^115^ ^]^ Copyright 2018, The Authors, published by AAAS. Reprinted/modified from ref. [115]. © The Authors, some rights reserved; exclusive licensee American Association for the Advancement of Science. Distributed under a Creative Commons Attribution NonCommercial License 4.0 (CC BY‐NC) http://creativecommons.org/licenses/by‐nc/4.0/.

In this context, SiNW arrays modified with a sugar‐responsive polymer containing phenylboronic acid (PBA) groups (termed SN‐PHB, **Figure** **8**a,i) combined three sequential functions: cell capture, intracellular delivery, and cell harvesting.^[^ ^143^ ^]^ First, specific recognition of PBA by the glycoproteins and SAs that span the lipid bilayer of the cell membrane enabled effective capture of both adherent (HeLa) and suspension (T and Ramos, Figure 8b) cells. Second, appropriate near‐infrared irradiation (NIR) was applied to exploit photothermal properties of SiNWs, facilitating efficient biomolecular (proteins and plasmid DNAs) delivery via membrane disruption (Figure 8a,ii). For hard‐to‐transfect T cells, flow cytometry detection demonstrated the significantly higher transfection efficiency (≈80%) of GFP reporter plasmids (pGFPs) using SN‐PHB under NIR irradiation (2.3 W cm^−2^, 30 s), compared with conventional lipofection (Lipo.2000) (Figure 8c). The captured cells were harvested under mild conditions by simply adding nontoxic sugar solution, due to the dynamic reversibility of boronated ester bonds between PBA and cis‐diol‐containing glycoproteins and SAs, allowing molecular exchange with higher‐affinity molecules such as fructose (Figure 8a,iii).^[^ ^146^, ^147^, ^148^ ^]^ The entire capture‐delivery‐harvesting process took <5 h, including a delivery time of <1 min. Such a multifunctional integrated SN‐PHB system, therefore, presents a simple yet powerful platform for efficient intracellular delivery with minimal impact on cells; this is highly desirable for biological research and future clinical applications.

#### 3.3.3 Microfluidic Devices Featuring VNS

VNS‐based platforms can be built in microfluidic devices to achieve massively parallelized, deterministic mechanoporation (DMP) for intracellular delivery into suspension cells.^[^ ^149^ ^]^ SiNWs (or penetrators) were specially designed and fabricated in confined wells (Figure 8d,e), where only a single cell can be captured and porated by impingement upon the penetrator under a negative aspiration flow (Figure 8f); cells were released by reversal of flow after which intracellular delivery occurs via diffusive influx of exogenous cargos (pGFP) through the single transient plasma membrane pore. Moreover, high cell viability and transfection efficiency were detected (both >87%) for DMP‐transfected Jurkat cells; mean pGFP transfection yield by the DMP device (88%) was over four times that by conventional BEP methods (20%) (Figure 8g). Efficient DMP‐mediated pGFP transfection of K‐562 (49%) and primary human T cells (82%) was also observed. This device overcomes the inherent stochasticity in many conventional membrane poration techniques, which usually results in a trade‐off between delivery efficiency and cellular viability. The massive parallelization allows high‐throughput cell engineering and rapid cell collection for subsequent processing, rendering a new means for addressing critical roadblocks in the manufacture and development of ex vivo cell therapies.

#### 3.3.4 VNS Coupled with Electroporation

Significant effort is now devoted to increasing the consistency of intracellular delivery on the basis of high efficacy and minimal cell perturbation. At the forefront of these efforts are the nanostructural‐electroporation platform‐mediated transfection technologies. Conventional BEP is widely used to transport diverse molecular cargoes into different cell types, but it is nevertheless stymied by limited fine control of membrane disruption, high rates of cell mortality, and inconsistent delivery outcomes.^[^ ^110^, ^114^, ^150^ ^]^ By contrast, nano‐electro‐injection systems (NES, Figure 8h–k) can focus localized electric fields at the nanostraw tips, significantly lowering the required voltage from kilovolts (typically >1000 V) in BEP to only a few volts;^[^ ^113^, ^114^, ^150^, ^151^ ^]^ this largely reduces damage to cells, cargoes, and electrodes during electroporation. Localized electric fields created by NES can transiently open pores on cell membranes, allowing electrophoretic delivery of bioactive agents without compromising major cellular functions.^[^ ^111^, ^115^, ^152^ ^]^ The combinatorial platform, by performing electroporation through nanostraws, allows a precise dosage control for cargo delivery in response to electric field modulation.

NES has demonstrated highly efficient delivery of dyes, plasmid DNAs, mRNAs, proteins, and Cas9 ribonucleoprotein (RNP) into a range of adherent cells and primary cells with >90% viability.^[^ ^115^, ^122^, ^153^, ^154^ ^]^ NES has also shown effective transfection on suspension Jurkat cells—a popular model for hard‐to‐transfect primary T lymphocytes;^[^ ^155^ ^]^ the net NES transfection efficiency (23.8%) was significantly higher than that of biochemical (11.2%), viral (19.5%), and electroporation (16.5%) methods.^[^ ^111^ ^]^ Analysis of cell doubling time, acute/chronic Ca^2+^ stress signals, and RNA transcriptomics revealed that NES resulted in lower Ca^2+^‐mediated cellular stress and negligible impact on cell division time. Moreover, NES exerted minimal perturbation on gene expression associated with immune cell activation and trafficking, compared with conventional viral and BEP methods. The intimate nanoscale cell–NES interface allows a drastic reduction in electroporation voltage required, maintaining higher cell viability. More importantly, this combinatorial platform highlights the advantage of creating uniform contacts between every cell and the source of the electrical pulses, reducing the variability of the local electric fields, so that a larger fraction of cells can be porated simultaneously at a specific voltage.

Apart from intracellular delivery, the scope for applying NES platforms has lately been broadened; they have demonstrated strong potential for nondestructive and longitudinal cell monitoring, attributed to their ability to manipulate cellular extraction/sampling and probe intracellular environment without compromising cell health.^[^ ^112^, ^154^, ^156^ ^]^ ZnO/Al_2_O_3_ nanostraws were decorated with numerous nanobranches (BNSs), which were conjugated with specific antibodies to facilitate effective capture of circulating tumor cells (CTCs).^[^ ^112^ ^]^ Artificial CTC blood samples were produced with MCF‐7 breast cancer cells spiked into healthy human whole blood. MCF‐7 cells overexpressed epithelial cell adhesion molecules (EpCAMs), and were separated selectively and efficiently (≈80%) by anti‐EpCAM‐coated BNSs from the blood samples. Moreover, the BNS‐based system facilitated both efficient delivery of biomolecules (PI dye ≥80%, GFP plasmids ≈70%) into MCF‐7 cells, and repeated extraction of intracellular enzyme caspase‐3 from the same group of cells; this allowed for real‐time and in situ regulation and monitoring of CTC intracellular activities, which is one of the most challenging aspects of cancer research. It is conceivable that this kind of technology will bring new opportunities to better trace and understand the functions of CTCs—including circulating malignant B cells in lymphoma patients—at different stages of a cancer.

### 3.4 Underlying Mechanisms of VNS‐Mediated Intracellular Delivery

**Figure 9:**
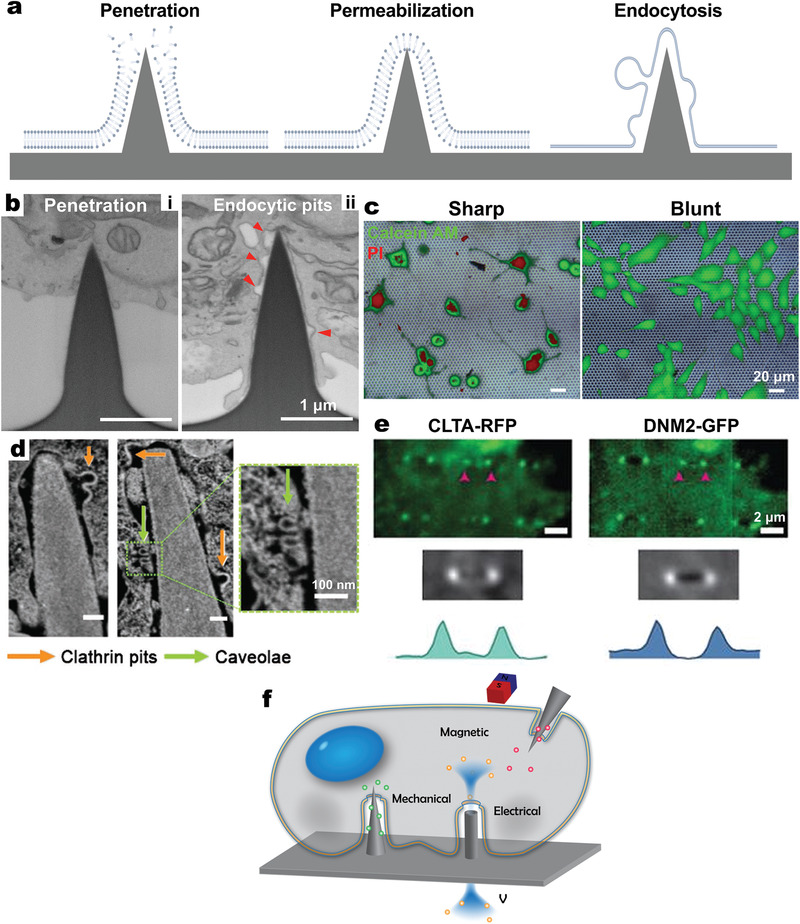
Mechanisms behind cell–VNS interactions. a) Schematic of molecular mechanisms behind the response of VNS‐mediated intracellular access and delivery, including mechanical penetration, membrane permeabilization, and endocytosis. b) FIB‐SEM images showing SiNW‐induced direct penetration into L1.2 cells (i) and endocytic pits in GPE86 cells (ii). b) Reproduced with permission.^[^ ^36^ ^]^ Copyright 2019, The Author, published by Wiley‐VCH. c) Fluorescence microcopy imaging showing the staining of PI (red) and calcein AM (green) to indicate membrane permeabilization and healthiness, respectively, of NIH‐3T3 cells cultured on “sharp” and “blunt” pillars (edge curvature radius: *R*_sharp_ ≈ 20 nm, *R*_blunt_ ≈ 250 nm). Reproduced with permission.^[^ ^164^ ^]^ Copyright 2018, ACS. d) FIB‐SEM images showing the accumulation of two types of endocytic vesicles, clathrin pits (orange arrows), and caveolae (green arrows), around nanoneedles. Reproduced under the terms of the CC‐BY Creative Commons Attribution 4.0 International license (https://creativecommons.org/licenses/by/4.0).^[^ ^116^ ^]^ Copyright 2019, The Authors, published by Wiley‐VCH. e) High‐magnification fluorescence images showing the distributions of CLTA and DNM2, two endocytic components involved in the clathrin‐dependent endocytosis, along nanopillars. Reproduced with permission.^[^ ^65^ ^]^ Copyright 2017, Springer Nature. f) Schematic of multiple mechanisms involved in the nanostructure‐mediated intracellular delivery. Reproduced with permission.^[^ ^165^ ^]^ Copyright 2019, ACS.

The exchange of cargos and signals across the cellular plasma membrane requires a collaborative interplay of diverse molecular mechanisms.^[^ ^157^, ^158^ ^]^ Rational design of VNS‐mediated intracellular delivery requires a deep fundamental understanding of these mechanisms. This has been the focus of a substantial research effort but there is still intense debate with respect to whether the mechanisms involve mechanical penetration, membrane permeabilization, endocytosis, or a combination of those effects (**Figure** **9**a).

#### 3.4.1 Mechanical Penetration

Under specific conditions, VNS access to the intracellular milieu is through spontaneous penetration of the cell membrane or alternatively under forceful nanoinjection. Published proof‐of‐concept work describes successful delivery via spontaneous penetration (cell piercing); but ongoing experimental and theoretical studies on the underlying penetration mechanism suggest that it is a rare event. The unresolved discussion behind cell piercing and its efficacy is underpinned by simultaneous modulation in multiple independent input parameters—such as using multiple types of interfacing scenarios, VNS geometry, cell types, cell stiffness and adhesion, and material composition and surface functionalization.

Several groups have found that spontaneous membrane penetration occurs when VNS geometry (density, length, and diameter) is optimized for a specific cell type.^[^ ^6^, ^28^, ^159^, ^160^ ^]^ For example, effective delivery of biomolecules into smaller nonadherent immune (naïve B and T) cells required SiNWs of length 2–3 µm, diameter <150 nm, and density 0.3–1 µm^−2^; slightly shorter and less dense SiNWs (1–2 µm; 0.15–0.2 µm^−2^) were favored for transfecting larger adherent immune cells (DCs and macrophages). These NWs were also reported to consistently penetrate cellular membranes without impacting cell health or morphology.^[^ ^28^ ^]^ But subsequent studies have yielded evidence against spontaneous penetration and suggested that most VNS fail to penetrate cells in the absence of external forces.^[^ ^131^, ^161^, ^162^, ^163^ ^]^ For instance, using a transparent CuO NW‐based cell impalement device and high‐resolution TEM, it was observed that the plasma membrane of HeLa cells wrapped tightly around the NWs, with the absence of spontaneous penetration.^[^ ^162^ ^]^

In a time‐resolved GFP quenching assay, hollow nanostraws of 100 nm diameter delivered Co^2+^ to quench GFP inside Chinese hamster ovary (CHO) cells. Detection of Co^2+^‐induced GFP quenching spots revealed that only 7% ± 3% of nanostraws were penetrated into CHO cells.^[^ ^136^ ^]^ But a strengthened cell–VNS interface—caused by either surface modification with adhesion promoters and/or application of active force—can significantly increase direct penetration events. For example, focused ion beam (FIB)‐SEM imaging has produced evidence of direct and frequent SiNW penetration into nonadherent L1.2 (mouse B) cells (Figure 9b, i), where appropriate centrifugal force was applied (200 g, ≈3.92 nN, 15 min).^[^ ^36^ ^]^ Ultimately, data from rigorous testing of the spontaneous penetration mechanism is still ambiguous, but has resulted in highlighting a unique set of combinations in which assisted VNS access and delivery is more likely to occur.

#### 3.4.2 Membrane Permeabilization

The bilayer is a permeability barrier that separates the cell from its exterior. Local cellular deformation induced by 1D‐VNS can cause lateral diffusion of lipids and transient membrane permeabilization.

Recent investigations suggest that the tight interface between 1D‐VNS and cell membrane is sufficient to induce local cellular deformation and subsequent permeabilization of lipids at the cell surface, which enables the direct access to the intracellular milieu.^[^ ^125^, ^164^ ^]^ By coarse‐grained molecular dynamics simulations of the cell membrane, researchers found that the high bending/curvature of the lipid bilayer particularly at the nano‐/micropillar tip can dramatically lower the traction force necessary to achieve membrane rupture.^[^ ^164^ ^]^ To verify the theoretical findings, they fabricated two types of pillars—“sharp” and “blunt”—with comparable diameter (2 µm) and height (2.5 and 1 µm) but different tip sharpness (radii of curvature: *R*_sharp_ ≈ 20 nm, *R*_blunt_ ≈ 250 nm). Impermeable dye propidium (PI) and permeable calcein acetoxymethyl (AM) dye verified membrane disruption and healthiness, respectively, of NIH‐3T3 fibroblasts cultured on sharp and blunt pillars. Cells remained viable on both pillars, as confirmed by the green calcein AM stain. But PI entered cells on sharp pillars with a probability of ≈70%, while no internalization by cells on blunt pillars was observed (Figure 9c).^[^ ^164^ ^]^ The experimental data supported the simulation results, indicating that accumulation of traction forces from high local deformation and tight focal adhesion increases transient permeabilization, which may allow rapid delivery of exogenous biomolecules into cytosolic compartment.

#### 3.4.3 Endocytosis

Endocytosis is the internalization of extracellular material. Endocytic pathways are an essential membrane trafficking process that use membrane‐bound vesicles as transport intermediators.

Given the complexity of nanotopographically induced membrane curvature artefacts—in particular, deformation and reorganization of plasma membrane (i.e., dynamic change in local lipid composition and curvature‐sensing proteins), the cytoskeleton remodeling, and nuclear envelope reshaping—a change in endocytosis behavior is likely to occur. Indeed, it is now increasingly evident that programmable surface nanotopographies on which a cell resides can trigger multiple independent endocytosis pathways—for instance, clathrin‐ and caveolae‐mediated endocytosis—accumulating caveolae and clathrin‐coated vesicles at the cell‐nanostructured interfaces, and in turn facilitating active biomolecular uptake without requiring direct penetration.^[^ ^116^ ^]^

By investigating VNS of different diameters (range 50 nm to 1 µm), recent studies have shown that VNS of diameter 50–100 nm are favored for receptor‐mediated endocytosis.^[^ ^36^, ^65^, ^116^, ^166^ ^]^ FIB‐SEM and TEM imaging have demonstrated VNS‐induced membrane curvatures and endocytic pits on adherent mouse fibroblast (GPE86) cells (Figure 9b, ii) and human mesenchymal stem cells (hMSCs) (Figure 9d), semiadherent neuroendocrine PC12M cells, and suspension Jurkat cells.^[^ ^36^, ^116^, ^163^ ^]^ Using fluorescence staining and confocal imaging, components of multiple endocytic pathways such as clathrin light chain, caveolin‐1, DNM2, and CLTA have also been observed to coassemble at highly curved cell membranes along VNS (Figure 9e).^[^ ^12^, ^36^, ^116^, ^167^ ^]^ An inhibition study treated GPE86 cells with nystatin and chloropromazine, finding that nystatin (which suppresses formation of lipid rafts, cholesterol‐enriched domains, and caveolae) caused greater impairment of Si nanotube‐mediated mRNA delivery than chloropromazine (which inhibits clathrin‐mediated endocytosis and interferes with fast endophilin‐mediated endocytosis).^[^ ^12^ ^]^ But for primary immune cells, especially for lymphocytes, endocytic pathways are often carefully gated via foreign element detectors such as TLR.^[^ ^168^ ^]^ It was reported that delivery of genetic agents using SiNWs did not activate endocytic or inflammatory pathways.^[^ ^28^ ^]^ Though PDMS pillars have been shown to promote F‐actin protrusion formation and lytic granule exocytosis of cytotoxic CD8^+^ T cells,^[^ ^33^ ^]^ it remains an open question whether and how membrane deformations and endocytic pathways are involved during VNS–lymphocyte interfacing. Because of their nonadherent nature, suspension immune cells interact less vigorously with VNS than do their adherent counterparts, making characterization of the interfacial interactions more difficult.

Due to the complexity of cellular responses toward physical, mechanical, and biochemical signals, it is highly likely that multiple pathways are invoked in VNS‐mediated intracellular delivery (Figure 9f, **Table** **1**).^[^ ^6^, ^8^, ^12^, ^14^, ^111^, ^125^, ^169^, ^170^ ^]^ A better understanding of regulation and crosstalk between different pathways would be extremely useful for designing advanced 1D‐VNS platforms to maximize transfection efficiency while inducing minimal cellular impairment.

**Table 1:** Summary of VNS platforms used for intracellular delivery in the literature

Type	Material	Diameter [nm]	Height [µm]	Pitch [µm]	Cell type	Reported delivery mechanism	Cargo type	Efficiency	Ref.
Nanowire	Si	100–200	≈3	–	HeLa, human fibroblasts (HFs), rat neural progenitor cells, rat hippocampal neurons	Spontaneous penetration	Plasmid DNA; siRNA; peptide; protein	>95%	^[^ ^5^ ^]^
		<150	1–3, >3	1–7	HEK 293T, primary immune cells (DC, NK, BMDC, macrophage, T and B cells)	Spontaneous penetration	siRNA	siRNA knockdown ≥69%	^[^ ^27^, ^28^, ^37^, ^118^ ^]^
		100–200	10–15	–	A549‐luc cells	Membrane perturbation	siRNA	–	^[^ ^125^ ^]^
		100	≈10	–	HeLa, Ramos, T cells	Membrane disruption (NIR irradiation)	GFP plasmid, (pGFP), RBITC‐BSA	>99%	^[^ ^143^ ^]^
		330–600	0.4–6.3	0.5–1.2	hDPSC, HeLa, HEK 293, HFF	Spontaneous penetration	pGFP	hDPSC, HEK 293, >85%; HFF, ≈61%; HeLa, ≈9%	^[^ ^159^ ^]^
		30, 90, 400	3–6	–	mouse embryonic stem cells, HEK 293	Spontaneous penetration	pGFP	<1%	^[^ ^160^ ^]^
Nanowire	ZnO	36.8	529.5	–	MCF‐7	Mechanical penetration (pneumatic pressure)	Molecular beacon	59.1%	^[^ ^128^ ^]^
Conical nanowire	Si	100	3.2	≈3	GPE86, L1.2, Jurkat, primary mouse T cells	Mechanical penetration (centrifugation), endocytosis	pGFP	GPE86, ≈22%; L1.2, ≈25%; Jurkat, ≈5%; primary mouse T, ≈30%	^[^ ^36^ ^]^
Nanoneedle	Si	≈200	>20	10, 30	NIH 3T3, HEK 293	Mechanical penetration (oscillation)	pGFP, Cre recombinase	pGFP, ≈34%; Cre recombinase, ≈42%	^[^ ^14^, ^131^ ^]^
Porous nanoneedle	Si	50 (tip) 600 (base)	5	2	HeLa	Mechanical penetration (centrifugation)	pGFP, siRNA	Codelivery >90%	^[^ ^8^ ^]^
					OE33, Het‐1A		QD	–	^[^ ^30^ ^]^
					hMSC	Endocytosis	siRNA	≈38%	^[^ ^116^ ^]^
Nanoneedle	Diamond	≈300	≈5	–	NIH 3T3, A549, hippocampal neurons	Membrane disruption (centrifugation)	EthD‐1, dextran, IgG, pGFP, QD, dsDNA	EthD‐1, ≈80%; dextran, ≈60%; pGFP, ≈40%; IgG, ≈35%; QD, ≈60%	^[^ ^13^, ^127^, ^169^ ^]^
Hollow nanoneedle	SiO_2_	250	5	5	NIH 3T3, HEK 293	Membrane permeation (saponin)	Dextran, plasmid DNA (pRFP)	Dextran, ≈70%	^[^ ^170^ ^]^
Nanostraw	Al_2_O_3_	≈150	1.5–2	–	Jurkat	Electroporation	pGFP	23.8%	^[^ ^111^ ^]^
		250	1.5	–	HEK 293, CHO	Electroporation	plasmid DNA (pRFP, pGFP), PI	Plasmid DNA, >67%; PI, >95%	^[^ ^122^ ^]^
		≈100	1000	–	CHO	Spontaneous penetration	Co^2+^ ions	6–12%	^[^ ^136^ ^]^
		100–200	2–5	–	CHO	Electroporation	Co^2+^, Alexa Fluor, pGFP	Co^2+^, 70%; Alexa Fluor, 40%; pGFP, 5–10%	^[^ ^152^, ^153^ ^]^
		150	1.5–2.5	–	HEK 293, hiPSC‐CMs, HSC, HFs, mouse primary glia cells, mouse primary neuron cells	Electroporation	mRNA (eGFP, mCherry), peptide (STIM1), Cas9 RNP	mRNA, 60–90%; Cas9 RNP, >90%	^[^ ^113^ ^]^
Nanostraw	Pt	400	1.5	–	HeLa	Electroporation	PI	>80%	^[^ ^154^ ^]^
Branched nanostraw	ZnO/Al_2_O_3_	300 (inner) 400 (outer)	1–2	–	MCF‐7	Electroporation	PI, pGFP	PI, 80%; ≥pGFP, ≈70%	^[^ ^112^ ^]^
Nanotube	Au	90 (inner) 180 (outer)	1.1	–	NIH 3T3	Optoporation	PI	≥95%	^[^ ^132^ ^]^
Nanotube	Si	300 (inner) 500 (outer)	1.5–2	5	GPE86	Mechanical force (centrifugation), endocytosis	IgG; ssDNA; mRNA; siRNA; Cas9 RNP	IgG/ssDNA, ≈80%; mRNA, >50%; Cas9 RNP, ≈15%	^[^ ^12^ ^]^
Nanofiber	Carbon	<100	10–17	5	CHO	Mechanical penetration (centrifugation)	shRNA, plasmid DNA	shRNA, >80%; plasmid DNA, > 60%	^[^ ^16^, ^117^ ^]^
Nanosyringe	Carbon	≈45	0.1–0.12	≈0.1	Primary lymphocytes	Mechanical force (centrifugation)	pGFP	≈46%	^[^ ^38^ ^]^
					HeLa, MCF‐7, PC3 MDA‐ MB‐231		siRNA	–	
					T47D, HepG2		siRNA, protein (GFP, RFP)	>70%	
Nanotips in microwell	Metallic	–	–	–	Ramos	Membrane poration (photothermal delivery)	Calcein green, dextran, pGFP, β‐lactamase,	Calcein green (0.6 kDa), >84%; FITC‐dextran (2000 kDa), >45%; pGFP, >58%	^[^ ^123^ ^]^
DMP device	SiO_2_	<1000	–	–	Jurkat, K‐562, primary human T cells	Penetrator impingement	pGFP	Jurkat, 88%; K‐562, 49%; primary human T cells, 82%	^[^ ^149^ ^]^

## 4 Concluding Remarks and Perspectives

Given their high programmability and biocompatibility, 1D‐VNS allow minimally invasive cellular manipulation. With their unprecedented spatial and mechanical resolution, 1D‐VNS surfaces have strong capacity to orchestrate ex vivo and in vivo immune responses. VNS‐based planar substrates and 3D microparticles stimulate innate immune cells and promote adaptive humoral and cellular immunity; 1D‐VNS demonstrate efficient intracellular delivery of a range of biomolecules into hard‐to‐transfect primary immune cells, overcoming the low efficiency and potential safety issues of conventional viral and nonviral methods.

Five critical challenges, however, remain to be explored. First, with the rapid expansion of multimodal cellular nanotechnology, developing new, cost‐efficient, and easily implemented nanofabrication routes will be essential to seamlessly integrating 1D‐VNS with biomedical research. Second, cellular processes at the cell–VNS interface are extraordinarily complex, due to the variability of immune cell types, diverse cargo types, as well as VNS surface functionalization, chemical composition, electronic properties, and physical geometry. So the crux will be to understand how such variability can be harnessed to maximize the degrees of freedom in which VNS‐mediated immune‐cell modulation and interrogation can be conducted. Third, most reports have solely focused on establishing successful delivery of bioactive cargoes, though as yet almost no progress have been made on assessing any adverse long‐term effects or comprehensively profile cell functionality and characteristics after VNS cellular perturbation. We anticipate that a closer convergence of experimental nanotechnology and immunology in combination with high‐throughput single‐cell RNA‐seq and new computational/bioinformatics methods will likely result in fundamental advances in immune‐oncology as well as for the validation of computational preclinical disease models. Specifically, codelivery of multiple types of bioactive cargoes with quantitative control is necessary if we are to open new research directions for engineering clinical‐grade CAR‐T and CAR‐NK^[^ ^171^ ^]^ cells in immunotherapy. For example, delivering into T cells a Cas9 RNP (to knock out surface antigens that might induce host rejection) together with a CAR gene would enable generation of a universal off‐shelf CAR‐T product; such a product would use T cells from healthy donors, avoiding reliance on low levels of effective T cells in patients. Fourth, combining powerful characterization technologies—such as cryoelectron microscopy, scanning ion conductance microscopy, and electron tomography—with apposite theoretical models will reveal detailed correlations of the synergetic parameters, enabling insights into the underlying mechanisms. Further advances in bioimaging will allow in vivo real‐time monitoring of molecular and cellular signaling pathways regulated by cell–VNS interactions. Fifth, cellular processes are natively dynamic, yet engineered VNS platforms are intrinsically static; so creating straightforward and efficient routes for dynamic cell manipulation, interrogation, and sensing coupled with analysing the cellular outputs in real time will have ramifications for mapping out and understating all levels of such nanobiointerfaces. A final central challenge in the field is to achieve the ability to scale these powerful nanoscale tools for clinical translation. Addressing these challenges will foster optimization and evolution of smart 1D‐VNS devices, paving the way for clinical and commercial translation of advanced cell‐based immunotherapy.

## Conflict of Interest

The authors declare no conflict of interest.

## Author Contributions

Y.C. and J.W. contributed equally to this work. The manuscript was written through contributions of all authors. All authors have given approval to the final version of the manuscript.
